# Underwater Optical Imaging Systems: A Comprehensive Review of Technologies, Architectures and Mission-Driven Design Trade-Offs

**DOI:** 10.3390/s26196127

**Published:** 2026-09-27

**Authors:** Dimitrios Piromalis, Christos Alexandris, Panagiotis Papageorgas

**Affiliations:** Department of Electrical and Electronics Engineering, University of West Attica, 12244 Athens, Greece; calexandris@uniwa.gr (C.A.); ppapag@uniwa.gr (P.P.)

**Keywords:** underwater optical imaging, marine imaging systems, light scattering, image restoration, underwater vision, computational imaging, remote sensing, polarization imaging, laser illumination, artificial intelligence

## Abstract

Underwater optical imaging plays a crucial role in marine exploration, ecological monitoring, underwater inspection, autonomous navigation, and scientific research, but it is heavily restricted by wavelength-dependent attenuation, scattering, refraction, and low visibility. This comprehensive review examines underwater optical imaging from a system-design perspective, integrating the physical principles of underwater light propagation with imaging modalities, illumination strategies, sensing technologies, computational methods, and system-level implementation considerations. A structured literature search was conducted using Scopus and Google Scholar, yielding 441 initially identified studies, of which 262 were retained after screening according to the defined inclusion and exclusion criteria, and seven additional recent studies were incorporated during revision, resulting in a final set of 269 publications. The reviewed literature is organized according to imaging modality and system architecture, including conventional RGB imaging, laser-based imaging, polarization imaging, multispectral and hyperspectral imaging, computational imaging, and AI-assisted approaches. Emphasis is placed on the relationship between environmental conditions, mission requirements, imaging range, illumination geometry, calibration, processing location, computational demand, and platform constraints. The analysis shows that no single imaging modality provides optimal performance across all underwater environments. Instead, effective system design requires matching the sensing and illumination strategy to water conditions, target distance, required image quality, available power, computational resources, and real-time requirements. The review therefore proposes a mission-oriented framework for understanding the principal trade-offs involved in the design and selection of underwater optical imaging systems and identifies adaptive acquisition, multimodal sensing, physics-informed AI, and optical–computational co-design as important directions for future development.

## 1. Introduction

More than 70 percent of the Earth’s surface is water, ocean, sea or lake, mostly still unexplored [1]. Despite today’s state of the art in imaging, it is not always possible to obtain clear images underwater. As easy as it might sound, acquiring underwater imagery is actually a difficult task [2], since the nature of water hinders optical underwater imaging. The ability to capture images of underwater environments or objects is useful for a wide range of applications, including but not restricted to environmental monitoring [3], underwater archaeology [4], pipeline inspection [5], and search and rescue missions [6]. Increasing interest in oceans and sustainability drives demand for higher-quality underwater imagery, especially that which can perform well in varying and harsh underwater environments [7].

However, optical underwater imaging is hindered by the behavior of light propagating in water [8] and is subject to absorption, scattering and attenuation of light in the transmission medium [9]. Some light wavelengths are capable of traveling deep into water as compared to other wavelengths. In fact, blue and green wavelengths penetrate deeper than red and infrared. Nonetheless, the attenuation of water is still quite high even at the blue and green light bands. Furthermore, the attenuation depends on the water quality, e.g., the concentration of dissolved and suspended organic matter, as well as the salinity of the environment [10]. As a consequence, the image becomes degenerated, which causes an improper color balance and reduced image details, contrast and visibility, thus making capturing and recovering high-quality underwater images challenging [3]. Therefore, significant attention is focused by researchers on hardware solutions such as light sources and lenses, as well as on algorithmic aspects such as image recovery and restoration through computer vision and artificial intelligence (AI) [11].

The underwater imaging field has expanded dramatically in recent years, from simple imaging to multispectral and hyperspectral imaging [12], stereo vision, laser imaging, and polarimetric imaging approaches [12]. Although each approach has its own benefits in terms of image quality, depth estimation, and light and color analysis [13], with digital sensors and image processing techniques, and with the recent advances in computational photography, the efficiency of imaging capture and processing has dramatically improved with real-time and automatic acquisition for ROVs (remotely operated vehicles) and AUVs (autonomous underwater vehicles) [14]. Additionally, by coupling optical imaging with sonar and acoustic data, the scope of underwater imaging has expanded into bathymetry surveying and 3D modeling of the underwater world [15].

The applications of underwater optical imaging are wide-ranging and increasing in importance. Applications include marine ecology: monitoring of coral reefs, coral reef health assessment, and mapping of coral reef biodiversity and habitats in noninvasive settings [8]; industry: underwater assessment of structures, inspection of underwater pipelines and cables, and offshore structural assessment [16]; archaeology: documentation of historical underwater sites and underwater archaeological structures; search and rescue: underwater surveillance, search, and detection and underwater object and target detection; and, more recently, environmental monitoring: water quality assessment (turbidity), underwater pollution monitoring, and underwater climate change monitoring [4,17]. With the increased attention to preserving the oceans and promoting sustainable use of the seas’ resources [18], underwater optical imaging and its importance are receiving increased attention.

The last few years have seen significant interest in smart systems that use machine and deep learning to achieve better object detectability [19], automated underwater target recognition, and real-time adaptation to varying water turbidities [20], as well as the availability of smaller underwater imaging systems which allow for easy deployment and are characterized by low energy consumption and the autonomous nature of systems [12]. Other efforts have aimed to develop bio-mimetic or multimodal systems that are capable of imitating biological vision systems, such as polarization vision of marine organisms [21], in order to improve the clarity of vision in turbid-water conditions and have explored the use of hybrid optical communication systems, which combine imaging, sensing and communication into a single unit [22].

Despite these research contributions, underwater imaging still poses a number of challenges, such as image degradation due to water turbidity [14], the reduced range of optical signals in seawater, the high cost of system maintenance [23], a lack of standard calibration protocols for underwater cameras that would allow inter-device comparisons, and a lack of standards for reporting in the literature [12].

In this paper, we provide an overview of the design of underwater imaging systems. We do so by considering different working principles, starting from basic physical concepts, then proceeding to the different architectures of systems and, finally, a comprehensive review. We first discuss the fundamental underlying concepts of underwater imaging, i.e., underwater light propagation, the attenuation effect, backscattering, and the different relations between different wavelengths, all optical effects that contribute to imaging quality in the underwater medium. In Section 2, we give some general background on how underwater optical imaging is carried out, including its potential and limitations. We devote Section 3 to discussing the architecture of underwater imaging systems. In Section 3, we review the different design parameters of underwater imaging systems, i.e., optical systems, sensor systems, and illuminating systems, and their effects on overall system integration, which is very important for imaging range, stability, and robustness to different underwater conditions. In Section 4, we introduce the review methodology as well as the approach taken for the data selection to ensure a comprehensive review process for the paper selection. This includes the reviewed literature, the databases used, the selection criteria, and the framework for categorizing the reviewed papers. Finally, we describe the approach used to determine the selected publications for inclusion in this paper and categorize them. Section 5 presents the findings of our review work and discusses the common patterns and typical architectures of underwater imaging systems that can be found for different application scenarios. In Section 6, we conclude the paper and propose future work and some areas for future exploration in underwater imaging, namely, adaptive imaging, multisensor systems, and onboard/AI-aided signal processing.

Previous reviews discussed different aspects of underwater optical imaging, such as general optical imaging and system development [24,25], underwater image enhancement [26], AI-based underwater object detection [27], and specific three-dimensional imaging in turbid media [28]. In contrast, the present review adopts an integrated system-design perspective in which sensing technologies, illumination, processing, communication, and system architecture are considered together. The proposed framework evaluates these choices according to mission objectives, water conditions, target distance, available energy, computational resources, and real-time processing requirements. This enables underwater imaging technologies to be compared according to their suitability for specific operational conditions and system-level trade-offs.

## 2. Underwater Optical Imaging Technologies and Applications

Underwater optical imaging refers to a set of technologies applied either to acquire or to monitor information related to underwater events/processes [29]. Over the years, underwater optical imaging systems have developed into complex systems mainly due to the great difference between underwater and above-water optical propagation [23]. Due to different underwater optical phenomena, modern underwater imaging systems can have more advanced hardware, e.g., sensors with better characteristics, specially designed underwater illumination systems, and post-processing algorithms, in order to cope with signal absorption, signal scattering, signal color distortion, and low contrast for good imaging quality [12]. In this paper, we briefly describe the different underwater optical imaging systems and their typical applications.

### 2.1. Light Propagation in Water and Its Implications for Underwater Optical Imaging

Underwater imaging quality in underwater images hinges on light transmittance. Underwater images tend to exhibit limited visibility, a restricted color spectrum, and low image sharpness in comparison with the air, which results from a reduced transmittance in the underwater medium [29]. Light experiences a series of phenomena upon entering water, such as absorption, in which the light is absorbed as it travels within the water [29,30]; scattering, where the light is scattered in another direction different from its original path; and, lastly, refraction, where the water medium has a refractive index that is different from that of air [29,31]. It is these three factors of attenuation, light absorption and scattering that have a significant impact on images acquired underwater as well as the intensity of the signals received [30].

The term absorption refers to the reduction in light energy due to the transport of light into the water medium as the energy in the light is absorbed. Therefore, as the depth increases in ocean water, the intensity of the light is reduced, and light penetration is also decreased [32]. Furthermore, as depth increases in the ocean, the water absorbs red and infrared light, while blue and green light become the dominant colors in underwater images [33]. Conversely, the water medium also contains different particles, for instance, biological organic matter and air bubbles. Thus, as the incident rays impinge on the particles, the light is reflected in other directions. This reduces the contrast of an image, and therefore causes the degradation of the image [34]. In summary, it is these three factors of attenuation, absorption and backscattering that are responsible for the degradation of underwater images and also impose a limit on the working range of cameras deployed in the underwater medium [35].

#### 2.1.1. Importance of Illumination Geometry and Light Angle

One consideration in illumination is the positioning of the illumination sources, as the position determines the incident angle of the light rays impinging on the object under investigation in the scene to be imaged [36]. This is because slight changes in illumination geometry can result in large variations in the quality of the final images, either deterioration improvement [37]. Positioning of the lights in very close proximity to the imaging device results in increased backscattering from the water, which degrades the quality of the image [38]. This is primarily due to the fact that light rays are backscattered from the water medium to the camera, where the water molecules and particles that reside in front of the camera backscatter light rays in the general direction of the image sensor, thus resulting in haze formation in the image [38,39].

Research on underwater light is particularly pertinent to this challenge. Even at extreme depths in pure water, light scatters significantly [3]. This was originally shown in papers such as “On the Penetration of Light into Sea Water” [40], in which the authors showed that as water depth and particulate density rise, the proportion of scattered light entering the camera’s field of view increases sharply, amplifying the veiling effects commonly seen in video imaging.

In more recent work, articles such as “Light availability in the coastal ocean: Impact on the distribution of benthic photosynthetic organisms and their contribution to primary production” [41] illustrate that light can easily become limiting in the coastal/turbid sea and emphasize the need for properly configured artificial illumination in these environments. Proper illumination becomes even more critical in scenarios where ambient light is low or rapidly attenuated by depth, as this helps alleviate the aforementioned veiling effects. The geometry of the light source as well as its illumination angle can be manipulated to reduce the degree of light scattering, so as to preserve the contrast and definition within the resulting image. To limit the amount of light that reaches the camera, light scattering, underwater imaging systems typically utilize off-axis illumination [42]. The term refers to illumination that is supplied from the side of the target [43] rather than the rear. In practice, this is achieved by positioning the light on one side of (and typically slightly higher than) the target so as to restrict the light that is observed by the camera to reflections from the target itself. This is especially vital for applications such as inspections or biological surveys, where the quality of images may be an essential requirement for the successful achievement of the project’s goals.

The angle of the light also changes how surfaces look. When light hits at a shallow angle, textures stand out more. Small cracks and details become easier to see. Shadows also look stronger [44]. But if the light is straight in front, things look flat. Depth is harder to notice. Details can get lost.

For video, this matters even more. Lighting needs to stay stable. If the angle keeps changing, the image can flicker. Contrast can shift. Sometimes visibility drops suddenly [45].

To formalize the effects described above, underwater light attenuation is commonly expressed using the Beer–Lambert law [46], which describes the relationship between absorbance and photon loss in a water column. In its logarithmic form, the law defines absorbance as

(1) $$A={log}_{10}\left(\frac{I_{0}}{I}\right)$$

where $I_{0}$ is the incident irradiance and I is the transmitted irradiance at depth. This formulation, widely applied in classical marine optics, provides a quantitative measure of how rapidly light intensity decreases in the water column due to both absorption and scattering processes [46,47]. Complementary scattering models incorporate the volume scattering function (VSF), which plays a central role in predicting backscatter levels that degrade underwater imagery, especially under artificial illumination [48]. These models form the physical basis for understanding contrast loss, veiling light, color degradation, and the strict illumination-geometry requirements discussed previously [49].

Beyond attenuation and particle-driven scattering, underwater imaging is further constrained by changes in the refractive index of seawater [50]. The refractive index (n≈1.33) varies with salinity, temperature, and pressure, and these variations introduce distortions that directly influence camera calibration and the fidelity of recorded imagery [50,51]. Empirical optical studies demonstrate that shifts in refractive index alter the optical path length and focal characteristics of imaging systems, leading to focal shift, apparent magnification changes, chromatic aberration, and geometric distortions at the frame edges [51,52]. Proper correction requires careful modeling of the camera–water interface, typically achieved using flat-port or dome-port housings combined with ray-tracing simulations that account for refractive transitions between air, glass, and water [53]. Contemporary underwater imaging research integrates these refractive index models to reduce distortion and maintain spatial accuracy across a wide range of depths and environmental conditions [54,55].

#### 2.1.2. Role of Artificial Lighting in Image Quality

Natural light does not last long underwater. It fades quickly, and as you go deeper, everything starts to look the same color. Because of that, artificial lighting becomes necessary if you want clear images [56]. Modern underwater lighting systems such as light-emitting diode (LED) CCD or CMOS arrays, strobes, or laser illumination are engineered to provide specific output in conditions where ambient light is insufficient [57]. These lights do not just make things brighter. They also bring back some of the lost colors and help keep video more stable across different depths and conditions [56].

But these systems have to deal with a few challenges:

- Colors fade with depth, especially reds, oranges, and yellows. Artificial light helps bring them back [9,43].
- Visibility can be very low. Strong, focused beams help make things clearer [58].
- Backscatter is always an issue. So the light has to be shaped and positioned carefully to reduce that fog-like effect [38,42].
- Conditions underwater are tough. Lights need to handle pressure, temperature changes, and buildup from marine life [59].

In many systems, like those used in ROVs, AUVs, or handheld cameras, lighting is just as important as the camera itself [60]. If the lighting is not set up properly, the image can end up washed out. Instead of seeing the scene, you mostly see particles floating in the water [3,61].

#### 2.1.3. Attenuation Models, Spectral Dependence and Sensor Detectability

Underwater signal degradation is dominated by wavelength-dependent absorption and scattering, which jointly control the effective imaging range and contrast of submerged optical systems. Light propagation in water is commonly modeled using a spectrally resolved exponential attenuation law derived from the Beer–Lambert principle:

(2) $$I\left(z,\lambda \right)=I_{0}\left(\lambda \right)e^{\left(-c\left(\lambda \right)z\right)},$$

where $I_{0}(\lambda )$ is the incident spectral irradiance at wavelength λ, I(z,λ) is the remaining spectral irradiance after propagation to depth z, and c(λ)=a(λ)+b(λ) is the spectral attenuation coefficient, consisting of the absorption coefficient a(λ) and the scattering coefficient b(λ). The propagation distance, z, is expressed in meters (m), while $a\left(\lambda \right)$, $b\left(\lambda \right)$, and $c\left(\lambda \right)$ are expressed in m^−1^. Wavelength, λ, is expressed in nanometers (nm). Recent ocean optics studies confirm that a(λ) increases sharply at longer wavelengths, while blue–green wavelengths (≈450–550 nm) exhibit minimum attenuation, explaining the preferential transmission of short wavelengths in underwater environments [62,63]. Beyond pure absorption, optical scattering strongly affects imaging performance. Forward scattering broadens the point-spread function of imaging systems, reducing spatial resolution. Backscatter creates a veiling radiance component that degrades contrast and introduces haze. These effects are described by the volume scattering function (VSF), β(θ,λ), which characterizes the angular redistribution of light by suspended particles. Recent experimental and modeling studies demonstrate that particle concentration and angular scattering profiles significantly influence underwater image quality, particularly under artificial illumination, where backscatter becomes the dominant noise component at short imaging ranges [9,64]. The radiometric signal reaching the camera sensor can be modeled as

(3) $$E_{sensor}\;\left(\lambda \right)=E_{S}\left(\lambda \right)+E_{bs}\left(\lambda \right)+E_{bg}\left(\lambda \right),\;with\;E_{S}\left(\lambda \right)=K\left(\lambda \right)E_{src}\left(\lambda \right)\rho \left(\lambda \right)e^{-c\left(\lambda \right)r_{s}}e^{-c\left(\lambda \right)r_{c}}$$

where $E_{s}\left(\lambda \right)$ is the target-derived signal spectral irradiance reaching the sensor, $E_{sensor}\left(\lambda \right)$ is the total spectral irradiance incident on the sensor, $E_{src}\left(\lambda \right)$ is the source spectral irradiance, $\rho \left(\lambda \right)$ is the target reflectance, $r_{s}$ is the source-to-target distance, and $r_{c}$ is the target-to-camera distance. $K\left(\lambda ,r_{s},r_{c}\right)$ represents geometric and optical-throughput factors, while $c_{w}\left(\lambda \right)=a\left(\lambda \right)+b\left(\lambda \right)$ is the wavelength-dependent beam attenuation coefficient. $E_{bs}\left(\lambda \right)$ and $E_{bg}\left(\lambda \right)$ are the backscatter and ambient/background spectral irradiance components, respectively. Separating direct signals and backscatter is consistent with established underwater image-formation models [65,66].

(4) $$N_{j}(r,\lambda )=A_{P}\tau E_{j}(r,\;\lambda )\Delta \lambda \eta (\lambda )\frac{\lambda }{hc_{0}},\;\;\;\;\;j\in \{s,\;bs,\;bg\}$$

where $j\in \left\{s,bs,bg\right\}$ denotes the target-signal, backscatter, and ambient/background components, respectively; $N_{j}\left(r,\lambda \right)$ is the corresponding detected photoelectron count; $E_{j}\left(r,\lambda \right)$ is the corresponding spectral irradiance incident on the sensor; $A_{p}$ is the photosensitive pixel area; τ is the exposure time; Δλ is the effective spectral bandwidth centered at wavelength λ; $\eta \left(\lambda \right)$ is the sensor quantum efficiency; h is Planck’s constant; and $c_{0}$ is the speed of light. This follows the standard irradiance-to-photoelectron conversion used in image-sensor models [67,68].

Detectability is governed by the signal-to-noise ratio (SNR), which can be expressed as

$$SNR(r,\;\lambda )=\frac{Ns(r,\;\lambda )}{\sqrt{Ns(r,\;\lambda )+N_{bs}(r,\;\lambda )+N_{bg}(r,\;\lambda )+N_{d}+\sigma_{r}^{2}+\sigma_{q,e}^{2}}}$$

with

(5) $$N_{d}=i_{d}\tau$$

where $N_{d}$ is the mean dark-current electron count, $i_{d}$ is the dark current in electrons per second per pixel, $\sigma_{r}^{2}$ is the readout-noise variance, and $\sigma_{q,e}^{2}$ is the quantization-noise variance expressed in equivalent electrons squared. Under Poisson approximation, the shot-noise variances associated with the signal, backscatter, background, and dark-current components are equal to their corresponding mean electron counts.

(6) $$r_{max}(\lambda )=\max\;\{r\geq 0\;:\;SNR\left(r,\;\lambda \right)\geq {SNR}_{min}\},$$

Accordingly, the maximum detectable range $r_{max}$(λ) at wavelength λ is defined as the largest range for which the calculated SNR remains equal to or greater than the required detection threshold ${SNR}_{min}$ [67,68].

Recent experimental and simulation studies confirm that the rapid spectral variation in $c_{w}(\lambda )$ directly constrains $r_{max}(\lambda )$, with red wavelengths becoming undetectable at shallow depths, while blue–green channels maintain usable signal levels at greater ranges [9,62,68]. These findings provide a quantitative basis for selecting illumination wavelengths, sensor types, and exposure strategies in modern underwater optical imaging systems.

In practical underwater environments, the Beer–Lambert attenuation term depends strongly on water type, because the attenuation coefficient is the sum of absorption and scattering contributions. Recent experimental/simulation-based studies report representative values that are widely used for system modeling: for clear ocean water, typical coefficients are $a\approx 0.114\;m^{-1}$ and $b\approx 0.037\;m^{-1}$, giving $c\approx 0.151\;m^{-1}$; for coastal water, $a\approx 0.179\;m^{-1}$ and $b\approx 0.219\;m^{-1}$, giving $c\approx 0.398\;m^{-1}$; and for turbid harbor water, $a\approx 0.366\;m^{-1}$ and $b\approx 1.824\;m^{-1}$, giving $c\approx 2.190\;m^{-1}$ [69]. Comparable environment-dependent attenuation values have also been adopted in recent underwater optical channel studies, commonly using $c\approx 0.15\;m^{-1}$ for clear water, $c\approx 0.4\;m^{-1}$ for coastal water, and $c\approx 2.19\;m^{-1}$ for harbor/turbid water [70]. These values immediately show why the imaging range collapses in turbid conditions: for example, at short stand-off distances the transmitted signal in coastal water decays far faster than in clear ocean water, and in harbor-like waters the signal can drop to negligible levels within only a few meters. Consequently, many recent studies explicitly model absorption and scattering coefficients by water type to predict the feasibility of optical sensing and transmission and to justify short-range imaging, stronger illumination, or alternative sensing modalities in high-attenuation environments [71].

### 2.2. Conventional Underwater Optical Cameras

Conventional underwater cameras have been the dominant technology used in most systems today [24]. Since these cameras offer easy image processing that can be implemented in most underwater systems like ROV, AUV, fixed and diver-based systems [25], they remain popular. Conventional underwater cameras typically share the same working principle as those on land, but modifications are usually applied in these systems to accommodate underwater environments. Conventional cameras are usually placed in pressure-tight housings [72] to withstand underwater pressure and corrosion from water, and they typically use special ports designed to adapt the camera systems [72].

Conventional cameras usually contain CCD or CMOS image sensors. In recent years, CMOS has become more widely used since it consumes less power compared with CCD, has faster processing speeds and also provides in-camera digital signal processing [72,73]. The type of sensor used in conventional underwater cameras depends on the application requirements; resolution, speed, sensitivity and power consumption are all important specifications that are taken into consideration [25]. High-resolution image sensors usually provide a more detailed image, which would be very useful for most inspections and measurements, although there are some cases where the low light conditions underwater due to water absorption prevent the image from being sufficiently well lit. A low-light condition usually has greater noise, which will require a larger aperture and longer exposure time [74]. Since a long exposure time and large aperture cause motion blur and greater noise, the image sensor must be chosen wisely [75].

Conventional underwater cameras usually include a set of lenses to achieve the required field of view, and these lenses are placed in a pressure-tight camera housing. The geometry of the lens in the system will change depending on whether a flat or a dome port is used, and it will introduce lens distortion [76]. Flat ports do, however, decrease the field of view and magnify the image [77]. Dome ports are more expensive and complex [78], although they provide better wide-angle imaging and are preferred for photogrammetry applications, since the optical lens geometry is unchanged. Since geometric distortion is commonly observed in underwater images from conventional underwater cameras and many underwater imaging applications will require quantitative measurements [13,79], it is important to perform a geometric correction on these images.

In practice, almost all regular cameras that are employed use artificial light sources and some edge image processing [80]. The issues with and the performance of underwater imaging are highly determined by environmental conditions but mainly by the parameters of the camera, e.g., exposure, gain, white balance and focus. Although in recent systems exposure and focus can be adjusted depending on the illumination conditions and distance to targets of interest, like in mobile robots [81], these features often malfunction underwater because of the poor image contrast and large scattering in the water column, and most industrial systems have fixed-focus cameras, or cameras are pre-calibrated [82].

Due to their flexibility, simplicity, cost and operational ease, traditional cameras are still very common and continue to serve as reference points to judge new underwater image processing algorithms such as laser imaging, polarized imaging and hyperspectral imaging [83]. Furthermore, conventional cameras are suitable for close-range observations and in clear water because of their good image quality and simplicity, low cost, and no added complexity in such circumstances [84].

### 2.3. Laser-Based Imaging Systems

#### 2.3.1. Laser-Line Scan (LLS) Imaging

Laser-line scan (LLS) imaging has recently attracted attention to improve underwater imaging, especially in low-visibility and turbid conditions [85]. Unlike conventional camera systems, LLS uses a laser to form a sheet of light which is used to illuminate the target surface. The camera then captures images reflected to it at an oblique viewing angle. With LLS moving continuously over a surface, lines of images are captured at regular time intervals, and the lines are stitched together to produce 2D or 3D images [86].

One big advantage of LLS is that it reduces backscatter a lot. Only a thin slice of water is lit at a time. So less scattered light reaches the camera compared to normal wide lighting [87]. This way, good-quality images can still be retrieved in turbid water with LLS. Therefore, LLS imaging is an optimal imaging method for applications which require high accuracy, for example, in pipeline inspections, bathymetric mapping and underwater infrastructure inspections.

However, such systems are not flawless; they necessitate an exact understanding of the object displacement [88], as well as keeping the distance between the laser and the camera constant [89]. The method can also be exposed to environmental vibration, leading to image distortions, as well as translational and non-linear movement errors of the platform [90].

#### 2.3.2. Laser Range-Gated Imaging (LRGI)

Another technique known to improve image quality is laser range-gated imaging (LRGI) [91]. Like all active sensing, LRGI relies on the use of a laser rather than ambient light [92]. LRGI uses a pulsed signal instead of a continuous signal [92]. This helps cut down backscatter problems.

An LRGI system produces images with higher contrast and with a greater effective range than images based on continuous or regular illumination [93], even in turbid waters where there is more light scattering [94]. Another benefit is that such systems capture a full image in one shot. Furthermore, because LRGI uses the entire camera, no mechanical scanning or translation is needed [92].

However, LRGI is a more expensive technology that requires greater resources [95], due to the deployment of a high-power pulse laser, fast-gated sensors/intensified cameras and electronic timing at a fine scale [96]. Thus, LRGI cameras are more expensive and have higher power consumption [97]. Moreover, LRGI images depend on the pulse width and the gate width, and the image quality diminishes as the pulse propagates in turbid water [98].

Recent developments also demonstrate the transition of time-gated and range-gated underwater imaging from laboratory configurations toward compact systems suitable for robotic deployment. A compact lock-in-enhanced time-gated imaging system designed for integration on ROV platforms has recently been validated in real-sea conditions under strong ambient-light interference, demonstrating the practical feasibility of active-gated imaging for field deployment [99]. Related work combining laser range-gated imaging with polarization-based backscatter suppression has further demonstrated improved target-image contrast under scattering conditions [92]. These developments indicate that compact integration, synchronization, power consumption, and overall size, weight, and power (SWaP) remain important system-level considerations when deploying gated imaging technologies on underwater robotic platforms.

#### 2.3.3. Other Laser-Based Techniques

In addition to LLS and LRGI, other laser imaging systems exist for use in underwater imaging, namely, LiDAR and laser speckle imaging. A LiDAR system determines the distance and reconstructs the 3D image of a water scene by measuring the time of flight and return intensities upon illuminating the water scene by laser pulses [100].

Such systems work best in shallow or fairly clear water, where the signal can still travel back without too much loss [101]. Compared to other scanning systems, LiDAR can measure depth directly and capture details better. But its capabilities are limited. Light still gets absorbed and scattered, so in murky water the ratio of accuracy to range drops dramatically [102]. Another method is laser speckle imaging. It uses the pattern created when laser light scatters and interferes. These patterns can tell us things like surface texture, movement, or flow [103]. This makes it useful for short-range studies or controlled experiments [104]. But its sensitivity is high compared to other imaging techniques and technologies. Small changes in the water, movement, or turbulence can affect the results [105]. For this reason, laser speckle imaging is not an effective method for producing large-scale spatial maps, but it is effective for analyzing the local small-scale dynamics close to a point of interest in stationary and controlled conditions [106].

To conclude, LLS, LRGI, LiDAR and laser speckle imaging are four methods with different characteristics, which may suit different applications in specific scenarios on a case-by-case basis, depending on the imaging objectives, such as imaging depth, turbidity level, the spatial resolution and the complexity of the imaging system.

### 2.4. Polarization Imaging

Polarization is another important parameter in underwater imaging. A polarization image, which measures the oscillation of light as it traverses through turbid water and interacts with the target, is different from a regular image which captures only the intensity of the light, i.e., the power incident in a given area of a surface [104]. When the light travels at some depth, its polarization is altered by a predictable mechanism of scattering that is well known [107]. Thus, polarization-based approaches are more suitable for underwater images, since they are able to reduce or even eliminate the backscatter effect and enhance the scene contrast in turbid water (see, e.g., [12]).

There are several advantages to using polarization cameras in underwater environments. The light that scatters from the particles is partially polarized [108], whereas light reflected from objects in the environment is polarized differently [108]; polarization cameras can filter out this scattered light. Polarization imaging requires special cameras that can capture information from multiple angles of polarized light either by rotating the filters or with polarization imaging sensors [109]. The result is clearer images, better edges, and stronger contrast compared to normal cameras [110,111].

Underwater, polarization imaging can be acquired via two different methods: either via temporal multiplexing, also known as “division of time” [110], which requires acquiring multiple images for several different states of polarization, or spatial multiplexing, also known as “division of focal plane”, where pixels are fabricated in the camera to sense different polarization states simultaneously [110]. There are a couple of ways to set this up. One way is to take images one after another with different filter angles. Although simple, one will struggle if things are moving [112,113]. Another way is to use special sensors that capture everything at once. These are better for “on-field” or “real-time” use, especially on moving platforms like ROVs and AUVs [114,115].

Spatially and time-resolved polarization cameras are more robust in poor-visibility environments and higher water turbidity [45,116]. They are used for object detection, navigation, studying marine life, and helping robotic systems see better [117]. Sometimes, they are combined with other methods or AI to get even better results [113].

But still, they have limits. In really murky waters, too much scattering can reduce the polarization effect [118]. The hardware is also more complex and can be expensive. Because of that, polarization imaging is usually used along with other systems, not on its own [118,119,120].

### 2.5. Hyperspectral and Multispectral Imaging

#### 2.5.1. Multispectral Underwater Imaging

Multispectral underwater imaging is the acquisition of imagery utilizing a restricted quantity of bands of color, typically restricted to the visible spectrum of the electromagnetic spectrum [121]. These systems are more sophisticated than traditional color cameras, though the disparity is not substantial; however, they yield added scene information [122]. This capability may be employed to perform seafloor classification, mapping of habitats, surveillance of coral reefs, and differentiation of features or objects on the basis of color [123].

As light attenuation is non-homogeneous throughout the marine environment, only the blue and green bands of light reach a substantial depth [124]. As such, these multispectral systems are frequently restricted to short ranges and may necessitate supplemental lighting of artificial light sources to produce a signal at desired wavelengths [124]. While such imaging does not always afford increased visibility, it is sometimes possible to yield additional scene details in close proximity [125].

#### 2.5.2. Hyperspectral Underwater Imaging

In addition to multispectral imaging, a hyperspectral imaging system gathers spectral information of many individual bands of wavelengths, in many cases up to dozens or hundreds of wavelength ranges [126]. This high-resolution spectral information permits the differentiation of fine-scale materials or biological features, thereby rendering hyperspectral imaging an attractive choice for a remote sensor of water quality, habitat mapping, or species identification [126,127].

As with multispectral imaging systems, hyperspectral imaging suffers similar light attenuation within the water column, and, in addition, most of the light spectrum is lost to attenuation as light wavelengths increase, leading to a poor signal-to-noise ratio [127]. Thus, these systems are typically restricted to short-range imaging and require not only bright illumination but also highly sensitive sensors [128]. Furthermore, the data output from hyperspectral sensors consists of large amounts of data that requires post-processing, storage and transmission [129]. Thus, hyperspectral sensors are usually used in conjunction with a variety of other imaging systems for the majority of applications; while hyperspectral sensors provide additional data layers in imaging systems, these are often not the primary systems employed for visibility of the site [130].

### 2.6. Computational Imaging and Artificial Intelligence

Artificial intelligence (AI) has become a big part of underwater image processing [27]. This is mostly because underwater images are often poor, due to light absorption and scattering. Almost always there is not enough illumination.

Instead of using fixed models, AI learns from data. It looks at degraded images and learns how to improve them. This makes it more flexible, especially in tough conditions where older methods struggle [27]. Deep learning methods can be deployed in a sub-surface vehicle or sensor, or on a ship, depending on the computing capabilities and the latency requirements of the given scenario [131].

#### 2.6.1. AI-Based Image Enhancement and Restoration

The task of image enhancement has recently drawn attention from the community that specializes in developing deep learning approaches for underwater imaging [132], where the objective is to restore image quality attributes such as structural details, texture, color fidelity, and visibility that have been distorted by different degradation factors that are present in underwater image acquisition scenarios [133]. These degradation factors are mainly caused by light attenuation and scattering in the underwater medium, uneven light distribution, and optical distortions due to aberration of optical system components. Unlike methods based on traditional models, such as physics-based models, deep learning approaches learn how to map degraded input to an enhanced output in a data-driven manner [134,135].

Convolutional neural network (CNN) models have been proposed to deal with underwater image restoration problems [136]. Various deep CNN models have been proposed to address various image enhancement problems, such as color correction, contrast enhancement, and dehazing. The aim is to mitigate backscatter and water absorption effects, recover color, and enhance edges and texture information [65]. Other architectures have also been used for underwater image enhancement recently, such as CNN-based, Transformer-based, and hybrid CNN–Transformer models, which can be used for learning long-range context and are thus less prone to non-uniformity in image degradation [137,138].

One big advantage is that many of these methods work on a single image. They do not need multiple views or extra data about the environment [139]. That makes them useful for real-time use or when working with old datasets [140]. Furthermore, video-based methods can take advantage of information contained across different frames to produce more temporally coherent outputs. This reduces visual artifacts such as flickering and often results in videos with less temporal noise. The coherence of enhanced videos may be especially relevant in cases when the application demands temporal consistency, for example, in automated inspection or moving-vehicle guidance tasks [26].

Despite all the advances that have been made using deep learning methods for underwater image enhancement, there are also several problems related to the quality and diversity of the datasets used [141]. The majority of datasets available have a limited diversity; the data usually only contains images acquired with one water type, depth, or lighting condition, which limits the capability of generalizing the developed methods to any given underwater scene [140,142]. Additionally, there might be domain shift if the AI models have only been trained with images from moderately turbid or even clear water and are applied to images that suffer from severe turbidity [134,143]. To tackle this issue, some researchers have used synthetic data, transfer learning, or fusion with physical light models in order to develop more physically grounded AI methods [143].

Another issue is balance. Some methods make images look nice but not always accurate [144,145]. Enhancement methods may add false image details (or may suppress components) that may be detrimental to later image analysis and scientific reasoning [146]. This can be a problem, especially in scientific work where accuracy matters more than appearance [66]. For example, in the case of image-based physical measurements, we may be interested in knowing if what we have measured is indeed correct in the light of an enhancement process. Due to these concerns, the field has also started to include metrics for enhanced image quality beyond visual inspection [147].

In real systems, AI enhancement is usually just one part of a bigger pipeline [140]. Sometimes it runs directly on the underwater device. This helps with quick decisions and better awareness, although it is limited by power and computing resources [140]. Other times, processing is done on the surface. This allows more complex models and better results. But it takes more time, since data has to be sent first [148]. So, there is always a trade-off: speed vs. quality. And that affects how these AI methods are used in real underwater systems [149].

Nowadays, research increasingly combines deep learning with physical underwater image-formation constraints. For example, Pham et al. proposed a deep unfolding framework that integrates physics-based underwater priors with learned regularization and contrastive learning, illustrating the growing trend toward hybrid model- and learning-based underwater image enhancement approaches [150].

#### 2.6.2. Depth Estimation and 3D Scene Understanding

Estimating depth and 3D structure is really important underwater [151]. It helps with navigation, avoiding obstacles, mapping areas, and handling objects. But underwater, this is not easy. Standard methods like stereo vision or active ranging do not always work well. Scattering, low-contrast, and limited sensor setups make things harder [152]. Because of this, AI has become a useful tool for estimating depth from images [153].

These AI methods usually use deep neural networks. They learn depth from visual clues, things like color fading, texture changes, shadows, and perspective [153]. Underwater, these clues are affected by how light behaves, so they can still give useful hints about distance [65]. With enough training, these models can estimate depth from a single image. They do not always need stereo cameras or special lighting setups [154].

There are different ways to train them. Some methods use supervised learning. They need real depth data, often collected using sonar, LiDAR, or similar systems [153]. These can be accurate, but collecting that data is hard and time-consuming [155]. Other methods use self-supervised or unsupervised learning. They look at patterns across video frames or multiple views [156]. These are more flexible, but they can struggle with motion blur, changing light, or unstable movement.

AI can also go beyond depth. It can help build 3D models, map spaces, and label parts of a scene [156]. This is useful for autonomous systems working in unknown areas. However, there are limits. If visibility is poor, or the image quality is low, the results will not be reliable. That is why these methods are often combined with image enhancement or other sensors [87,90].

Another issue is generalization. A model trained in one type of water might not work well in another [157]. Models trained in one water type or illumination condition often exhibit degraded performance when deployed in different environments [65]. Variations in turbidity, lighting geometry, and camera calibration introduce domain shifts that are difficult to capture fully during training [66]. Recent research efforts aim to address these issues through domain adaptation techniques, multimodal learning, and the incorporation of physical priors related to light propagation and camera geometry [143].

Computational demand also affects deployment strategy. Lightweight models are more suitable for onboard, latency-sensitive tasks such as navigation and collision avoidance [148], whereas computationally intensive depth reconstruction and 3D modeling are more commonly performed at the surface or during offline post-processing [158,159]. Consequently, practical systems may adopt hierarchical processing architectures that separate real-time estimation from higher-complexity reconstruction.

#### 2.6.3. Object Detection, Classification and Semantic Understanding

The detection of objects and semantic understanding with AI approaches is another rapidly growing approach for underwater imagery interpretation [140]. But underwater this is not easy, because images often have low contrast and as a result colors look off. Objects can be partly hidden. And many things look similar, especially marine life or structures [65]. Because of this, detection and classification are harder than on land. Deep learning has helped a lot here. These models learn patterns directly from underwater images, instead of using fixed rules [160].

Most systems are based on convolutional neural network architectures, which originally were first made for normal, land-based images [161]. They are then retrained using underwater data. This helps them detect things like marine animals, structures, debris, or seabed features [162]. Classification models are also used. They help identify species, group habitats, or detect unusual patterns. This is useful for environmental monitoring, inspections, and security tasks [161].

There is also semantic segmentation. This goes further. Instead of just finding objects, it labels every part of an image [163]. This helps in tasks like seabed mapping, coral reef studies, or checking structural damage [151]. But this is harder to do underwater. There is not much labeled data, and the environment changes again and again [164]. This demands strategies like data augmentation, weakly supervised methods and transfer learning [165].

Moreover, AI-driven semantic understanding remains essential for supporting autonomous navigation decisions [158]. By combining detection, classification, and segmentation outputs, autonomous underwater vehicles can identify obstacles, track objects of interest, and adapt mission behavior in real time [159]. Such systems promise enhanced efficiency through lowered intervention needs and mission duration [166], yet real-time inference is currently constrained by onboard hardware limitations, power constraints and available bandwidth [149].

A key challenge in underwater semantic understanding is model robustness and generalization [157]. Variations in water clarity, lighting conditions, sensor characteristics, and biological diversity can lead to significant performance degradation when models are deployed outside their training domain [65]. To address this issue, recent research has investigated domain adaptation, multimodal fusion with acoustic or inertial sensors, and self-supervised learning strategies [143]. The goal is to make these systems more flexible and reliable across different underwater conditions [136].

#### 2.6.4. Information Quality Assessment and Entropy-Based Evaluation

The advent of AI-based approaches in the domain of underwater image enhancement and analysis has highlighted the importance of objective, quantitative image quality assessment [142]. As manual or subjective analysis will be inappropriate for autonomous or big data applications, image processing algorithms should therefore be evaluated in terms of how much information they preserve or recover [144]. Such evaluations can be based on information theory [167]. Information content is often quantified using entropy, which assesses the amount of information in an underwater image prior to and following image processing [168]. In this context, entropy measures the distribution of image pixel intensities or features, with higher entropy indicating more texture, contrast and structural information [146,167]. AI-based enhancement methods often aim to maximize information content by recovering attenuated edges and suppressing noise, and entropy measures provide a convenient means of comparing different algorithms or parameter settings without reliance on reference images [147].

Global entropy has been used to assess the information content for the full image. However, several more advanced approaches have also been proposed to quantify local entropy, mutual information and structural similarity indices for spatially non-uniform and semantic evaluation [144]. For example, mutual information is important to evaluate the extent to which the original scene structure has been preserved in the enhanced image and prevent the appearance of artificial features or loss of physical meaning [146]. Information quality evaluation has been increasingly applied in underwater image analysis to assess the trade-off between visual appearance and information preservation [142].

Information quality assessment also plays a critical role in adaptive processing pipelines [144]. In autonomous underwater systems, real-time estimates of image information content can be used to dynamically adjust processing strategies, such as selecting between lightweight enhancement and more computationally intensive restoration methods [169]. Similarly, entropy-based metrics can inform decisions related to data compression, transmission priority, and storage, ensuring that limited communication bandwidth is allocated to the most informative visual data [149].

Despite their utility, entropy-based and information-theoretic metrics have limitations [147]. High entropy does not necessarily correspond to improved task performance, particularly when noise amplification or over-enhancement occurs [146]. Consequently, recent research emphasizes combining information metrics with task-driven performance measures, such as detection accuracy or classification confidence, to provide a more holistic evaluation of AI-based underwater image processing [142]. This integrated assessment framework is especially important for mission-critical applications where visual quality must directly support autonomous decision-making [149].

Recent underwater image quality assessment methods have moved beyond conventional handcrafted metrics toward learned perceptual models. URanker introduces a ranking-based no-reference underwater image quality assessment framework that learns relative perceptual quality using a convolution–attention Transformer [170]. More recently, SCUIA has employed semantic contrastive learning and domain adaptation to improve underwater image quality assessment across different underwater environments. These approaches highlight the increasing importance of learned and domain-robust quality assessment for evaluating underwater image enhancement methods [171].

#### 2.6.5. Computational Complexity, Processing Speed and Future Directions

The practical deployment of AI-based underwater image processing systems is strongly constrained by computational complexity, processing speed, and energy consumption [166]. Deep learning models have improved a lot in underwater imaging [160]. They help with things like image enhancement, depth estimation, and scene understanding. But there is a problem. These models need a lot of computing power. And most underwater systems do not have that kind of capacity. Because of this, speed becomes very important. Especially for real-time tasks. Things like navigation, avoiding obstacles, and making decisions on the spot all depend on fast processing [148].

Underwater platforms such as AUVs and ROVs typically operate with limited onboard computing resources and strict power budgets [66]. High-capacity graphics processing units (GPUs) or specialized accelerators may be impractical due to size, heat dissipation, and energy constraints [166]. Consequently, many AI-based imaging pipelines adopt lightweight network architectures, reduced model precision, or task-specific optimizations to achieve acceptable inference times [169], though this may involve an inevitable trade-off between processing speed and enhancement quality or semantic accuracy [159].

Latency considerations also influence how processing tasks are distributed between underwater systems and surface or edge processors [172]. Time-critical operations, including basic image enhancement and obstacle detection, are often executed onboard to ensure immediate responsiveness [173]. More computationally intensive tasks, such as high-resolution restoration, multiframe fusion, or large-scale semantic analysis, are typically deferred to surface-based processing, where greater computational resources are available [174]. This hierarchical processing strategy reflects a broader trend toward distributed perception pipelines in underwater robotics [172].

Recent research has increasingly focused on reducing the computational cost of AI-based underwater imaging [175]. Model-compression techniques such as pruning, quantization, and knowledge distillation can reduce memory and computational requirements, while hardware-specific optimization can improve inference efficiency with embedded accelerators [176]. These approaches are particularly relevant to real-time and long-duration autonomous deployments, where computational throughput and energy consumption are tightly constrained [166].

Real-time underwater AI nevertheless remains challenging because image quality, communication bandwidth, and environmental conditions can vary substantially [65,149]. Adaptive processing strategies therefore seek to balance inference speed, model complexity, and image quality according to the operating conditions [172,177]. Such trade-offs are particularly important for long-duration autonomous missions with limited onboard resources [166].

The values in Table 1 are reported values from the respective studies and should not be interpreted as direct rankings because the methods were evaluated using different datasets, input resolutions, hardware platforms, and experimental protocols. Full-reference metrics such as PSNR and SSIM, no-reference underwater image quality metrics such as UIQM and UCIQE, and task-oriented metrics such as mAP therefore quantify different aspects of performance. Inference time and model size are included only where explicitly reported by the corresponding study. Directly comparable energy-consumption measurements were not reported across these representative studies and are therefore not estimated here.

### 2.7. Mission Profiles and Design Implications

The design of underwater optical imaging systems is largely determined by the mission objectives rather than the imaging mode itself [181]. All fields have different demands in terms of imaging distance, turbidity, spatial resolution, frame rate, sensor and platform size and mobility, and power supply [11,12], which leads to the choice of the light source, camera, and post-processing calibration and method [58]. As a design community, we have to see ourselves as responding to a mission-oriented requirement rather than providing a sensing solution [182].

Other applications are those that involve marine ecology and habitat observations, such as using a short-to-medium-range camera to image either shallow water or water that is somewhat clear where true color and consistency over time is of importance [100,108,183]. These applications can include various short-term diver-deployed platforms or more permanent installations on either fixed or mobile structures that allow for the collection of time-series datasets [184,185]. Some important considerations are the stable geometry of the illumination, low power demands, and the ability to repeat calibration [3,37]. The trade-off between spectral richness versus power and storage requirements is usually easily satisfied by conventional RGB cameras or by RGB cameras supplemented with additional bands [38], with the main consideration for these applications being proper positioning of the illumination, perhaps artificial, to avoid backscatter. Computational enhancement is frequently applied in post-processing rather than onboard, reflecting the non-real-time nature of many ecological studies [89,186]. The dominant trade-off in this domain lies between spectral richness and power or data storage constraints [23].

Other applications are very different from the previous examples and include applications for industrial inspection and subsea infrastructure assessment [20]. These applications typically are imaging at close range (e.g., 1–3 m) in turbid (harbor or coastal) waters [38,104]; capturing detailed structural information is essential because inspection is often done with the use of remotely operated vehicles (ROVs), and the ROVs are usually tethered, thus making the power requirements of the systems less of an issue [13]. Off-axis, high-intensity illumination becomes essential, and in highly scattering environments laser-based techniques such as laser-line scan or range-gated imaging may provide superior performance compared to conventional cameras [87]. Robust geometric calibration and mechanical stability are required to support defect detection and measurement tasks [73,115]. Image processing may be required on board vehicles to provide feedback to a human operator or for some other reason, though most computationally intensive processing is done in post-processing [182]; the trade-off in these applications is generally between the implementation complexity of the hardware and how robust it is and the quality of the underwater images [3,45,70].

For robotic perception and autonomy, the system must be able to run in real time on a platform in motion. AUVs, generally, are limited in their power supply and computational resources, and it is necessary that imaging is performed in real time to support real-time decisions such as obstacle avoidance, bottom-following, or target-tracking. Also, the depth of the field is often limited and the environment could change depending on depth and water turbidity [187]. Therefore, it is usual to utilize lightweight RGB or stereo cameras and AI algorithms running on board to solve such problems. For instance, polarization imaging, or even illumination-based methods, can be utilized when the contrast is heavily compromised due to water scattering. More advanced solutions like hyperspectral or laser-based active imaging methods are less frequent as they typically require high power and are hard to implement [43,56,59]. For underwater autonomous navigation, the primary design compromise is to balance the robustness of the perception system and the real-time compute it requires, which means choosing algorithms that balance computational speed and accuracy [188].

3D point-cloud accuracy is required in both geometry and radiometry to correctly reconstruct the geometry and preserve the site for future inspection [4]. Typically, this use case operates at short range or in a controlled environment where images can be obtained from divers or from an ROV from overlapping viewpoints [189] for structure-from-motion algorithms [189]. Accurate calibration of camera intrinsics, optical port geometry, and lighting uniformity is essential. While conventional RGB cameras are commonly used, dome ports and precise calibration procedures are often necessary to minimize refractive distortion [13,115]. Computational enhancement is typically performed offline, where high-resolution reconstruction and dense point-cloud generation are prioritized over latency [12]. As a result, the main compromise here is usually the ease of use against the quality of the reconstruction.

For the underwater environment, now, monitoring of a habitat or water quality is rarely used only for visualization but instead to classify different types of particles, pollutants, and biogenic particulates [190]. This can be achieved with the usage of multispectral or hyperspectral cameras for close-range applications where the differential attenuation of the wavelengths is somewhat controlled [3]. However, spectral cameras usually demand a lot of effort in terms of calibration, data acquisition and processing and might limit some applications. As a result, they are mostly found in fixed-deployment setups or for research purposes where higher energy and computational use is acceptable. The main compromise is thus between the spectral features of a sensor and its usage [3,61].

Across all domains, the relationship between mission requirements and design choices can be generalized as follows: shorter imaging ranges allow simpler hardware but demand careful illumination geometry [191]; increased turbidity shifts the design preference toward structured or gated illumination [3,192]; long-duration deployments prioritize power efficiency and stable calibration [193]; and real-time autonomy constrains algorithmic complexity [194]. These mission-driven relationships form the organizing principle for interpreting the imaging modalities discussed in previous sections [191]. Rather than representing competing technologies, conventional cameras, laser-based systems, polarization imaging, hyperspectral sensing, and AI-driven enhancement constitute a toolbox of design options, each optimized for specific environmental and operational conditions [28].

Because there are so many options, it helps to compare them clearly. The next part (Table 2) brings everything together. It shows how different systems work, where processing happens, and what trade-offs they involve. This makes it easier to see how these technologies fit into real underwater applications. This framework enables a concise visualization of how acquisition modality, processing distribution, real-time feasibility, and computational complexity interact within practical underwater systems.

From Table 2, we could infer the following: most conventional RGB imaging systems are used in distributed systems, with an onboard camera and offboard processing, and usually have lower computational complexity and lower image acquisition requirements, which could easily result in performance degradation under degraded optical conditions. Laser-based imaging systems usually provide better contrast and a larger imaging range, with relatively uniform performance, but usually more computing power and greater energy consumption are needed for data processing, which requirements are difficult to achieve in real-time operation onboard an underwater vehicle. Polarimetry or multiwavelength imaging systems usually provide additional information to the scene structure, and they can be used to decompose certain components of scenes. However, they are more difficult to deploy in practice, due to higher requirements in terms of system calibration and higher imaging speed, as well as a higher data rate, and are typically applied in lab environments and off-line post-processing. Artificial intelligence (AI)-based systems are more robust and flexible to the changes in different imaging scenarios, with onboard real-time enhancement and scene understanding, such they are more suitable for deployment in autonomous scenarios. However, high computational power and power supply are generally bottlenecks for their deployment in autonomous vehicles. Ultimately, there is no “best.” It is not a matter of just which sensor or which algorithm works well; you must think about the operational distance, the computing platform, and what the end user wants the device to actually do. Thus, looking at a table like Table 3a showing range, backscatter, resolution, and complexity will provide you with more information about how the techniques interrelate.

Table 3a indicates that no single imaging modality simultaneously maximizes operational range, backscatter rejection, spatial resolution, and implementation simplicity. Conventional RGB systems offer relatively low hardware complexity and real-time acquisition capability but remain sensitive to turbidity and backscatter. LLS and LRGI provide greater backscatter rejection and effective ranges at the cost of increased power demand, geometric or timing sensitivity, and system complexity. Multispectral and hyperspectral systems provide richer spectral information but require greater acquisition, calibration, data-handling, and computational resources. Polarization imaging can improve contrast under scattering conditions, whereas AI-based enhancement can improve perceptual or semantic interpretation without overcoming fundamental limitations in the acquired optical signal. Modality selection should therefore be treated as a system-level design decision based on working range, turbidity, power availability, platform motion, calibration requirements, and real-time processing constraints. The references associated with each imaging modality in Table 3a also constitute the source set used for the corresponding qualitative synthesis in Table 3b.

Parameters such as exact power consumption, data rate, and system cost are not consistently reported across the reviewed literature and therefore were not assigned quantitative values where direct cross-study comparison was not supported.

The decision matrix appeared in Table 4 is intended as mission-oriented engineering guidance rather than as a set of fixed selection rules. The suitability of each imaging modality depends on the combined effect of environmental conditions, required working distance, platform motion, available illumination, power budget, computational resources, and real-time constraints. Consequently, modality selection should be performed at the system level, with sensing, illumination, calibration, processing, and platform capabilities considered jointly rather than independently [181]. In practice, hybrid or multimodal configurations may be preferable when environmental conditions vary substantially during a mission or when a single sensing modality cannot provide sufficient robustness across the required operating envelope.

## 3. System Design and Processing Architectures for Underwater Optical Imaging

As a result, we move towards a more general architectural level of analysis and move beyond the comparison of individual modalities. In Section 2 we listed the pros and cons of different sensing methods, including their operational envelopes, but a specific sensing method is only useful as part of a system that takes into account processing requirements, storage requirements, and transmission requirements, as well as the requirements that are specific to the operational environment, the existing hardware and the communication link. This section takes an underwater optical image and data acquisition, transmission and processing systems approach to this specific underwater image acquisition system as it interfaces with underwater image pre-processing, transmission and surface post-processing. In Section 2, we presented an overview of underwater optical image technologies and data processing methods for such data. We have yet to present the design of such systems, which determines how and when images will be processed, stored and transmitted. The choices made here, for example, determine if pre-processing and post-processing of the underwater images take place underwater or on the surface and how computational and communications systems constraints will be met, how an underwater image system will interface with other subsystems on the underwater platform or remotely operated vehicle, etc. In general, the system design of underwater image systems can be thought of as a series of data processing pipelines (and associated communications links) which take image acquisition, pre-processing, transmission and post-processing to be generic functional blocks of an end-to-end image acquisition system. By using a system-design point of view, we will discuss some typical underwater optical image architectures found in the underwater optical image processing literature we presented in our survey by going over typical underwater image pre-processing, underwater and surface image post-processing as well as transmission system capabilities. By modeling these systems in general terms as a series of data processing and communication pipelines, we will introduce a framework for examining and comparing the different system-design trade-offs for different implementations of such systems. By doing so, we can compare system design across application areas, vehicle platforms and operational environments.

### 3.1. End-to-End Underwater Image Processing Pipeline

Through our overview of the different underwater optical image technologies available, the image processing algorithms that can be utilized for underwater imagery and the different system implementation designs from related work presented in Section 2, we have already been able to identify common image processing pipelines and their associated data flows. While in practice these systems might be very different as far as the specific image acquisition sensor and vehicle platform are concerned, as well as their overall complexities, often they share common image processing capabilities and operations. Specifically, the systems in question generally utilize the same basic operations of image acquisition, pre-processing, transmission and post-processing. In general, by abstracting away the image processing steps common to each system, we can identify end-to-end underwater image acquisition, pre-processing, transmission and post-processing pipelines across different system implementations. Figure 1 presents a block diagram depicting a typical system for common system-design patterns; more specifically, this is a representation of an end-to-end underwater image acquisition, underwater image pre-processing, transmission, and post-processing system. In reality, this is not a single system but a representative of many of the systems presented in our survey.

Figure 1 depicts a fundamental and illustrative pipeline for underwater imaging systems, illustrating the trajectory of image data from acquisition beneath the surface to display and analysis above. This framework is intentionally generalized to ensure applicability across diverse systems; it highlights the broad data flow rather than any particular hardware or algorithmic realization. Components of this pipeline are subsequently elaborated upon in Section 3.2, Section 3.3, Section 3.4, Section 3.5, Section 3.6 and Section 3.7. Below, each element of the pipeline is examined in greater detail, with specific attention paid to the influence of each stage on the overarching underwater imaging architecture. The initial phase, underwater image acquisition, entails gathering scene luminance with optical lenses and transforming that luminous information into electronic signals via image sensors and analog-to-digital converters [199]. As the entry point into the system, this phase dictates the quality and character of incoming image data and is governed by environmental factors such as lighting geometry, light scattering, and light attenuation [200]. Following acquisition, the data advances to an underwater image pre-processing step encompassing operations such as demosaicing, denoising, light normalization, and a preliminary degree of color correction. Generally, these operations are kept to the bare minimum to enable data transmission rather than image enhancement [188].

Subsequently, the data is passed on to the transfer and storage step. This phase accommodates the requisite link bandwidth and storage space, but also includes data framing, organization, metadata attachment (such as timestamps, camera, and sensor data) and compression, depending on available system and link parameters [182,188]. Finally, this pre-processed and organized data is routed to the underwater communication interface linking the underwater and surface systems both electrically and logically [182]. Various platform options exist for realization of this interface, for instance, fiber-optics, Ethernet-over-tether, power-line communication, or even acoustic or optical wireless channels [182,201].

Subsequently, a communication interface at the surface retrieves the bitstream and distributes it to other surface devices [200,202]. Since a wide range of computationally intensive post-processing algorithms (e.g., higher-order color correction, image dehazing, super-resolution, 3D reconstruction, or deep learning-based analysis) exist [202,203], they may be performed by a user at the surface once the bitstream is transferred. The reconstructed images may then be displayed, saved to a storage device, or analyzed further. Consequently, post-processed images may be useful for visual inspection, archived data records, and automated processing [203].

This scheme may be viewed as a two-step framework with two separate processing stages, with the first occurring on the seafloor and the second on the surface. Such a division of processing is a significant design decision that allows the system designer to balance image quality, processing delay, available system computation power, and communication bandwidth to best suit the mission. This design is also flexible, since the pipeline stages may be optimized independently, and one does not influence the other [9,22,68,115,204,205,206,207,208].

### 3.2. Underwater Image Acquisition

Now that the general framework of the pipeline is established (in Section 3.1), we consider each of the pipeline components in depth. The image acquisition process is considered first, as it consists of more than just a single stage. This process consists of two distinct steps, which are optical in nature and electrical in nature, subject to different constraints and considerations during the system design. Considering the problem from this perspective will give further insight into how the environment, sensors, and operating conditions work together and how they contribute to the raw images that are used by the remaining pipeline stages.

In Figure 2, an enlarged view of the acquisition block can be seen, depicting almost the entire optical and electrical chain in the capture of radiance from the underwater scene to the digital image that is subsequently processed. This formulation uses multiple acquisition models described in related underwater image research [9]. They describe that underwater images capture radiance from underwater scenes and both natural and man-made illumination [9]. In a natural underwater scene, radiance decreases significantly with increasing depth; therefore, most underwater cameras use an artificial illumination source, such as an LED [9], strobe [56] or laser [9,43,59]. Most previous work emphasizes the importance of the illumination and most commonly uses off-axis illumination [43,56,59]. This is one of the reasons the illumination system is a sub-component of the underwater imaging system, as the position and distance of the lights to the camera are also important [3,21,22,61,98,110,114].

The illuminated scene is observed through the water optical path, where absorption and scattering distort the spectral and spatial distribution of light before it reaches the camera [209]. Numerous optical studies demonstrate that the water column acts as a wavelength-selective and range-dependent filter, strongly constraining achievable imaging distance and color fidelity [210,211,212]. These propagation effects motivate both the placement of artificial lights close to the camera and the use of short-range imaging geometries in practical systems [29].

Light entering the camera passes through the housing and optical port, which forms the refractive interface between water and the internal air-filled camera volume [213]. The difference between flat ports and dome ports affects the distortion and the focal-length shift of the system. Choosing the wrong port for an imaging system will negatively impact the resolution and the field of view in an underwater image [58,76,77,78]. The optical port design is typically one of the most critical considerations when building an underwater camera, particularly for applications requiring a wide field of view and for metric reconstruction applications [214,215].

The light then transmits through the camera optics, where the resulting image projects onto the image sensor. Here, photons generate electric current within the sensor itself [67,216]. CMOS image sensors are commonly used in underwater applications since they have low-power requirements, they can run at high frame rates, and can generally be integrated into smaller devices [217]. Because an underwater image sensor sees a lower light level reaching the sensor itself, the relevant image sensor specifications to be considered for such applications would be the sensor’s quantum efficiency, noise characteristics, and dynamic range [218].

The sensor output is conditioned by the analog front end (AFE), which applies gain, black-level correction, and noise suppression prior to digitization [219]. The analog front end is crucial for maximizing the effective dynamic range of the scene being imaged. It is often run with an automatic exposure control in order to maximize the effective dynamic range of the scene [81,220]. Next, this signal is sent to the ADC and timing/synchronizer to produce the digitized signal. The timing and synchronizer also provide the timing needed for the image sensor as well as for image/illumination synchronization [206,221].

The last step in the imaging system chain is the raw frame buffer. This section holds the resulting digital image data as raw frames and passes these frames into the appropriate image processing algorithms [222]. It is worth noting that a subset of the data in the raw frame buffer can be accessed to then be used to control the exposure, gain, and illumination of the sensor, effectively a form of feedback to help ensure the stability and quality of the imaging system regardless of the range and/or turbidity in the scene to be imaged [223]. This describes a general imaging system configuration found in most contemporary underwater imaging systems and is vital in providing the necessary input for downstream image processing systems [81].

### 3.3. Underwater Image Pre-Processing

The pre-processing pipeline illustrated in Figure 3 represents the typical sequence of lightweight image operations performed underwater prior to data transmission. Unlike surface post-processing, which may involve computationally intensive enhancement or semantic analysis, underwater pre-processing is designed to be resource-efficient and robust, focusing on stabilizing raw sensor data and preparing it for reliable communication [224].

The pipeline begins with frame validation and synchronization, which ensures temporal consistency across image sequences and verifies frame integrity before further processing [45]. This stage is particularly important in tethered and wireless systems, where dropped frames or timing misalignment can propagate downstream and degrade reconstruction or analysis performance [224].

Next, sensor-level corrections are applied to compensate for hardware-induced artifacts, including dead-pixel suppression, black-level adjustment, and demosaicing [182]. These operations establish a consistent baseline image representation and prevent sensor noise from being amplified by subsequent processing stages [225].

The noise reduction block addresses low-light and high-gain artifacts that commonly arise in underwater imaging due to limited photon availability. Simple spatial or temporal filtering methods are typically employed at this stage to improve signal quality while maintaining low computational overhead [226].

Following noise suppression, basic color compensation and white balancing are performed to partially correct wavelength-dependent attenuation and illumination non-uniformity. These adjustments are intentionally conservative and do not attempt full-color restoration, which is generally deferred to surface processing where more advanced algorithms can be applied [227].

The dynamic range adjustment block normalizes brightness variations caused by changes in target distance, turbidity, or illumination intensity. By compressing or stretching intensity distributions, this stage improves visual consistency and prevents saturation or underexposure across consecutive frames [203].

Optional resolution scaling or cropping may be introduced to adapt data volume to available bandwidth or to prioritize regions of interest [228]. This design choice reflects a trade-off between spatial detail and transmission efficiency, particularly in bandwidth-constrained deployments [81]. The resulting images are embedded in the message payload, in addition to other auxiliary data such as system clock, platform status, and acquisition details [229], and delivered to the interface in a format well suited to provide readily available data to the higher-layer functions responsible for image processing and data decoding [222].

These pre-processing steps ensure system robustness, efficiency, and flexibility [230]. Outputs from this stage can also be routed back to regulate the image acquisition parameters and thus close the control loop, allowing the system to operate at its optimum performance (e.g., exposure, gain, and illumination levels). To minimize the overall system complexity while still preserving image quality at the surface, the underwater pre-processor provides the information needed to perform image processing and higher-level functions such as image enhancement/analysis and scene understanding.

### 3.4. Underwater Data Formatting and Compression

The underwater data formatting and compression function block transforms the image frames produced by the underwater pre-processing block into a data stream suitable for transmission via the underwater communications link while balancing video quality, responsiveness, and link throughput [231]. In the first step, the frames are organized into the data format specified by the underwater communications protocol and then combined with timing, imaging system configuration and platform status details to assist the decoding and synchronization procedures [231]. Optionally a color-space conversion (RGB to YUV) may also be performed to reduce overall data volume or emphasize luminance data over chrominance in order to limit link bandwidth requirements [232]. Second, image and video compression techniques are employed to further reduce the size of the compressed video data to fit the link capacity, delay and platform capability constraints [232,233].

From a design point of view, this block of Figure 4 is essentially the balancing act between image quality and connectivity bandwidth. Since underwater systems have severe limits on the data rate, power, and energy consumption, a compression scheme is required [234]. Many systems use feedback from the transmission buffer to adjust how data is sent. Things like bitrate, quality, or frame rate can change on the fly. This helps keep things stable when the connection conditions change [235]. At this stage, compression is usually kept light. The goal is not to shrink the data as much as possible. It is more about keeping things stable and fast. If compression is too strong, it can damage the image and make later processing harder [235]. So, this step is more about making the system work smoothly, not perfect optimization. The idea is to keep the important visual details while still allowing real-time performance.

### 3.5. Underwater Communication Interface

The underwater communications interface provides a link between the onboard image processing system and the data analysis on the surface. Depending on the system for which the underwater communications interface is used, this interface can be implemented very differently. In tethered systems, i.e., ROV systems, an Ethernet or fiber-optic-based high-bandwidth communication link is often used to transmit low-latency, high-quality video [199,236]. In a hybrid system, the image data and power can be transferred in the same tether using power-line communications or in an untethered AUV system wirelessly using acoustic or optical underwater communication links, both of which provide much less available bandwidth, which can highly fluctuate [237]. Due to the variety in implementation, it is not possible to consider the underwater communications interface as a unique element for all underwater imagery systems. Instead, it is part of the specific design of a system and dependent on the system type, the range of the system, and the requirements of the mission [237].

Prior to transmission, formatted image data as appeared in Figure 5 is staged in a transmission buffer where rate control and scheduling decisions are applied. Depending on the communication technology in use, a link adaptation layer may adjust parameters such as bitrate, packet size, or redundancy based on feedback from the surface receiver or estimated channel conditions [238]. In bandwidth-constrained systems, this adaptation often propagates upstream, triggering changes in compression strength, frame rate, or spatial resolution within earlier processing blocks [202]. Consequently, the communication interface acts not merely as a passive transport channel but as an active architectural component that influences the overall imaging pipeline, mediating trade-offs between image quality, latency, and robustness under varying underwater communication conditions [237,238].

### 3.6. Surface Image Post-Processing

Surface image post-processing is the final step in the imaging pipeline, where the image data is received and reconstructed so that it can be used. On the surface, it is the phase when the received data is decoded and the packets are reassembled into a frame; it is also when the system tries to correct or compensate for any errors caused by data corruption [239]. Next, the images may be improved, further processed, and displayed or sent on to other systems for display by an operator. The reconstructed image frames are buffered before any processing is performed so as to establish a clear separation between the communication subsystem and the image processing subsystem that follows later [240].

If underwater image pre-processing is mainly concerned with effectiveness and robustness, surface image post-processing as appeared in Figure 6 is a computationally demanding procedure that includes dehazing, color correction, super resolution, and multiframe fusion [241]. These processes exploit the greater computational resources available at the surface or edge servers and aim to maximize visual clarity and information content [240]. Surface image post-processing utilizes the extra computational resources that are available at the surface (or the edge) and attempts to obtain an image that is close to the original. The image post-processing system may also implement artificial intelligence (AI)-based image processing algorithms like object detection, semantic segmentation, depth estimation and so on to support infrastructure inspection and ecological survey applications [189]. Finally, the restored image may either be shown on a screen or stored for later post-processing [242].

In the design framework, the second step closes out the imaging loop, feeding data back into earlier stages. Not only does it acquire data, but it is also able to learn from the acquired data; for example, data from the optical surface receiver like channel state information can be used to adapt transmission methods. Likewise, AI data can inform data acquisition in this step (e.g., frame rate, resolution, and illumination). Instead of an additional distinct step, this is part of the imaging loop that ties the sensing, communications, and analysis together as a closed-loop adaptive system to improve performance for different scenarios.

### 3.7. Automated and Manually Operated Underwater Imaging Systems

Modern underwater optical imaging systems can be broadly categorized according to their operational paradigm: automated platforms, where image acquisition and processing are driven by onboard control logic, and manually operated systems, where capture parameters and platform motion are largely controlled by human operators [243]. In this paper, the distinction between manual and automated systems is important because choosing one or the other will have significant implications on the overall system design, from the integration and pre-processing methods and the communication system requirements to the application of feedback [244].

#### 3.7.1. Automated Operated Imaging Systems

Automated system workflows use off-the-shelf cameras as part of a fixed platform system or as an autonomous vehicle payload based on the standard operating procedure with imaging geometry that is either static or fixed, and instead of performing manual acquisition, images are offloaded to be processed [186,245]. Automation is utilized for monitoring and measuring tasks including but not limited to length measurements utilizing stereo 3D reconstruction and structured imaging for mapping tasks where the reproducibility of the baseline, observation viewpoint, and time is important for obtaining scientific measurements [246,247].

##### FLIR Camera Systems

Teledyne FLIR (FLIR) sensors are well suited to underwater scientific engineering applications in which performance needs to be predictable, the timing precise, and the hardware modular [248]. The availability of industrial interfaces such as GigE Vision and USB3 Vision (and of reliable radiometry) is a primary draw of FLIR cameras in system design [249]; these are needed to ensure both system-level integration with lights, guidance and navigation sensors and that synchronization can be implemented effectively. As a result, FLIR cameras are frequently included in sensor-heavy ROV and sled deployments for benthic habitat mapping, in monitoring of structures, and in marine biological studies in which repeatable and recalibrated observations are needed [248,249,250].

FLIR cameras are configured not to process the image in camera, but rather to deliver a raw, or lightly processed, image to capture software [251]. This allows the software to control how the captured image will be edited, compressed or AI-analyzed [185] to suit the needs of its application. A drawback of using FLIR cameras, though, is that the overall system complexity of the ROV increases; when deploying an FLIR camera underwater it must also have a custom enclosure, custom window, and external lighting attached. Overall, the choice of an FLIR camera is appropriate when the flexibility and controllability of the system is prioritized over the price, size and system complexity of the final ROV [185,251,252].

##### Open-Source Underwater Imaging Systems

Open-source underwater imaging systems have gained increasing attention as flexible and cost-effective alternatives to proprietary platforms, particularly for research-driven deployments and rapid prototyping. Systems such as those described in [253,254,255] demonstrate how openly documented hardware and software stacks enable low-cost biological monitoring, time-series imaging, and in situ ecological observation. In a similar way, they show that open robotic platforms can be adapted for specific tasks. Users can change how images are captured and processed to fit what they need.

From a design point of view, these systems focus on flexibility. They are built in a modular way, so parts can be adjusted or replaced. They often use standard computing boards and camera sensors, combined with custom housings and user-controlled lighting.

We can see this approach in [253,254,255]. In these systems, users can directly control things, like when the camera captures images, how the lighting works, and how data is collected. This makes it easier to connect sensing, onboard processing, and communication in a more controlled way. In contrast to closed commercial platforms, these open designs allow direct access to sensor parameters and synchronization mechanisms, making them well suited for experimentation with adaptive imaging strategies and task-driven perception. This flexibility is further exploited in software-centric frameworks such as that reported in [256], which operates on externally acquired imagery to support customizable detection and tracking pipelines.

Another important contribution of open-source platforms lies in their role as experimental testbeds. Systems such as SeeStar [253], PlasPi [254], IPAX [255], and the OpenROV-based lionfish detection platform [257] are routinely used to validate new acquisition concepts and AI-based perception methods under real underwater conditions. At the same time, broader benchmarking and review studies such as [181,258] highlight how open datasets and openly described workflows accelerate comparative evaluation of enhancement and reconstruction techniques across institutions.

Despite these advantages, open-source underwater imaging systems also introduce design trade-offs. As shown across SeeStar, PlasPi, In situ Plankton Assemblage eXplorer (IPAX), and the OpenROV-based assistive system, reliability, long-term sealing, and operational robustness are largely dependent on user-implemented solutions rather than industrial-grade engineering. Because of this, these platforms are usually used for short missions or in controlled settings, or for testing ideas. For longer or critical missions, more robust systems are preferred, such as the ROV stereo vision system presented in [187]. At the same time, review and benchmarking studies help show what these systems can and cannot perform. There is also a clear gap between controlled environments and real-world conditions. And that difference affects how imaging systems are designed and evaluated. Underwater imaging performance differs fundamentally between controlled environments, such as swimming pools or test tanks, and natural seawater environments. In controlled pools, water clarity is high, optical properties are spatially uniform, and suspended particulate matter is minimal, allowing image formation that closely approximates ideal laboratory conditions [259,260]. Because of this, things like camera calibration, 3D reconstruction, and color consistency can be done quite accurately in controlled settings [260,261]. But real seawater is very different. Light gets absorbed in different ways depending on the wavelength. There is also scattering, and the water can be uneven in clarity. All of this reduces contrast and distorts colors, especially as distance increases [247]. On top of that, there are other factors. Marine life, particles in the water, and uneven lighting all affect the image. These problems usually do not exist in controlled environments [14,262]. So, systems that work well in pools or lab conditions often do not perform the same in the open sea. This shows that design and testing need to consider real ocean conditions from the start [224].

#### 3.7.2. Manually Operated Imaging Systems

##### GoPro

The GoPro is a small, commercially available, waterproof device capable of producing high-quality videos [263]. From a design perspective, its primary advantage lies in enabling dense visual coverage at low cost, which makes it particularly suitable for multicamera rigs, stereo configurations, and photogrammetric surveys [186,264]. Many studies show that GoPro-based systems can work well underwater, if they are set up properly [265]. With good camera placement and enough overlap between images, they can produce useful results. Things like 3D models and habitat maps are possible, especially in shallow and fairly clear water.

But there are some limits. GoPro cameras do not give much control because the camera lenses are fixed and exposure settings are limited, not to mention the fact that a lot of the processing happens inside the camera automatically [266]. This can make calibration harder and affect color accuracy. Because of that, getting good results depends on how the system is set up. Factors like proper lighting, stable mounting, and capturing the same area from multiple angles all help. Post-processing is also important to fix color issues and compression effects [267]. Thus, it is not really about the camera alone. What matters more is how it is used as part of the whole system [184].

##### DSLR-Based Underwater Imaging Systems

Digital single-lens reflex (DSLR) cameras are widely used in underwater scientific imaging when high optical quality, large sensor size, and precise control over imaging parameters are required [213]. From a design perspective, their primary advantage lies in the availability of interchangeable lenses and full manual control over exposure, aperture, and focus, enabling consistent radiometric behavior across image sequences [268]. These characteristics make DSLR-based systems particularly well suited for applications such as underwater photogrammetry, habitat mapping, and high-resolution documentation of benthic environments, where geometric accuracy and image fidelity are critical [184].

The deployment of DSLR cameras underwater typically relies on dedicated pressure housings and carefully selected optical ports, with flat or dome ports chosen based on lens characteristics and field-of-view requirements [213,269]. While these systems can deliver superior image quality compared to compact or action cameras, they impose significant design constraints related to size, weight, and mechanical complexity [215]. In addition, limited frame rates and storage capacity can restrict their use in long-duration or real-time missions. As a result, DSLR-based underwater imaging systems are most commonly employed in diver-operated surveys or short-duration ROV missions, where controlled acquisition conditions and post-processed analysis are prioritized over continuous real-time operation [246].

In Table 5 and Table 6 we focus on technologies, imaging modalities, applications, lightining and image acquisition. Table 6 focuses in the environment each platform and vehicle operates in, the fundamental limitations, design bottleneck, and the processing method. 

## 4. Comprehensive Review Methodology

In this review, we take into account the literature on the subject of system design, sensing techniques, optical setups, technologies, and applications in the field of underwater optical imaging. The search in Scopus and Google Scholar databases was carried out within a period of approximately two months from 2 February 2026. These databases were chosen as they are rich in material related to engineering, oceanography, photonics, computer vision, and underwater imaging.

A variety of keyword phrases grouped together based on the topics of interest in this review were used in the search query. General terms such as “underwater optical imaging” and “underwater vision” were used. Terms regarding systems such as “optical system design” and “sensor configuration” were also used. Propagation-related terms like “attenuation,” “scattering,” and “turbidity” were incorporated. Technology-related terms such as “laser imaging,” “polarization imaging,” “multispectral imaging,” “hyperspectral imaging,” and “fluorescence imaging” were utilized. Application-related terms like “marine ecology,” “seabed mapping,” “ROV imaging,” “AUV imaging,” and “underwater navigation” were also included. In addition, topics on computational aspects of underwater imaging, such as underwater image enhancement, restoration, artificial intelligence, and image quality assessment, were explored. No strict lower publication-date limit was imposed. Greater emphasis was placed on the recent literature, while earlier publications were retained when they were considered seminal, foundational, or necessary for describing fundamental optical principles and established underwater imaging technologies.

The literature search initially identified 441 publications. Following relevance assessment and subsequent screening according to the inclusion and exclusion criteria described below, 262 studies were initially retained for comprehensive review; however, 7 additional recent articles were later integrated to ensure a more comprehensive overview, bringing the final total to 269. The database-specific search approach, search fields, search expressions, search period, and retrieval results are summarized in Table 7. The combined search and supplementary identification process initially yielded 441 potentially relevant publications. Additional seminal and relevant studies were identified through backward and forward citation searching of key publications.

The literature search initially identified 441 potentially relevant publications. Following screening of titles and abstracts, 373 publications were considered sufficiently relevant to the scope of the review. A second screening stage removed duplicate publications, substantially outdated studies that did not provide foundational or still-relevant information, and publications that offered limited technical contribution to the objectives of the review, resulting in 301 publications. The remaining papers were then assessed in greater detail against the defined inclusion and exclusion criteria, including their relevance to underwater optical imaging, availability of technical or experimental information, and contribution to system design, optical configuration, or practical deployment. Following this assessment, 262 publications were retained for inclusion in the review. After revision, seven more studies were included in the literature review, bringing the total number to 269as shown in Figure 7.

## 5. Discussion

The reviewed studies indicate that underwater optical imaging design should begin with the mission requirements and environmental constraints rather than with the selection of a particular imaging technology. Short-range operations in clear water can often be addressed using conventional RGB or stereo imaging with careful calibration and illumination. As turbidity and scattering increase, controlled illumination, polarization, laser-line scanning, and range-gated imaging become more advantageous, although at the cost of increased power consumption, calibration requirements, hardware complexity, and integration effort. Missions requiring real-time autonomous operation further constrain sensor choice and algorithm complexity because onboard power and computational resources are limited. Conversely, applications allowing offline processing can prioritize higher resolution, spectral information, and computationally intensive reconstruction. Thus, the appropriate imaging architecture results from balancing environmental conditions, required range and image information, real-time constraints, power availability, computational resources, and platform limitations.

From a review of the literature, it is apparent that the field has evolved. Many of the older works presented individual components of a system (such as a specific image processing technique), and these were often not discussed within the context of the imaging system’s design. Prior work has looked at imaging underwater from a physics perspective and from an imaging system architecture perspective; we combine both of these ideas to focus on system design. The aim here is to understand the overall influence of the type of sensor, the type of lighting arrangement, and the image processing techniques being used, particularly how these components work together in a specific context, on underwater imaging. Our work is based on paper [29] in large part due to its physical grounding and meticulous use of physics-based modeling, while older papers such as [34,37] are still fundamental to our discussion due to the insights into underwater imaging that are presented therein. Reference [66] is another rigorous work on system design which couples radiative transfer theory with engineering; we believe this is of high technical merit in a way that no other work is. This is of relevance since the illuminator of the system is often a secondary detail for engineers. Reference [66] provides a very detailed physical modeling of the underwater imaging system that is used to evaluate the depth and turbidity of an underwater environment. Its inclusion here is very important as it shows how a combination of imaging setup geometry, light placement relative to the camera, and light wavelength can be used to improve signal-to-backscatter ratios. However, our focus is not on using the deep learning method as a post-processing improvement, but rather to show that the design of the system itself needs to be the central factor that drives the imaging system. The findings of this paper serve as the technical backbone for the design-focused approach advocated in this survey.

In Section 2, it was established that underwater optical image performance is limited by how light travels through the medium, with attenuation and backscatter defining the range and contrast limits of the imaging systems [38,40,46], showing that even modest changes in constituent composition can substantially alter attenuation and backscatter behavior, which directly explains why imaging solutions do not transfer cleanly across “clear,” “coastal,” and “harbor” conditions. Moreover, a recent literature review [32] emphasizes the need for better system conditioning to enable high-quality measurements and imaging during underwater surveys; the authors attribute a major portion of the reasons for poor image quality in the literature to poor environment characterization (or assumptions of its properties) as opposed to the quality of the image processing algorithms.

Beyond bulk attenuation, Section 2 highlights that illumination geometry is a decisive design variable in underwater optical imaging, often exerting greater influence on image quality than sensor resolution alone. Studies examining backscatter-suppression strategies demonstrate that source–camera separation, angular offset, and structured illumination can substantially improve contrast in scattering-dominated regimes. For example, [192] shows that modifying the spatial relationship between illumination and imaging axes directly reduces veiling light without requiring post-processing compensation, while, similarly, structured and laser-based approaches, including implementations discussed in [90], exploit controlled beam geometry to confine the measurement volume and thereby limit scattering contributions from out-of-plane particles. As the results described here imply, the selection of a lighting source geometry to match the camera FOV is critical to image improvement and optimization of the solution. In order to ensure the best imaging performance, the optical system design (light + sensors, geometry and FOV) must be carefully considered prior to any imaging solution deployment. Too many of the current imaging systems suffer from low SNR and contrast, due to lack of a properly optimized optical system and insufficient sensor and light conditioning, leading to sub-optimal performance and the subsequent development of poor-quality imagery.

Methods of image formation using spectral and polarization measurements use additional data, other than the intensity of the scalar signal, from the properties of light to distinguish object reflectance from scattering. Various physical models capturing the properties of polarization in marine environments exist in the optical oceanography community, such as work published by Mobley in [39], which describes the scattering for different polarization angles and the resulting geometry of the polarized field. This physical description was applied by Schechner and Karpel’s paper referenced as [36], showing that the backscatter term can be removed and the object’s contrast improved using polarizing filters if the angle of the illumination and transmission axes were changed appropriately. While this article is over twenty years old, it has remained a popular and heavily cited work in establishing the possibility that polarimetric data could provide additional cues to improve the contrast of objects in scattering media.

Recently, polarimetric data has also been employed for applications such as navigation and geolocalization [40,41]; however, it still suffers from the same challenges, such as alignment sensitivity and configuration changes, as other methods relying on additional cues like range images, thermal images, or depth data. Overall, spectral and polarimetric imaging methods are significantly more complex than the methods that only utilize intensity or contrast; while their additional information content offers potential benefits for their respective tasks, they also require that the imaging systems account for additional physical effects that limit their ability in turbid waters. Neither the spectral nor the polarimetric signature of the target can solve problems associated with the effects of scattering in turbid waters. Instead, they merely move these problems elsewhere to additional tasks, such as the need for complex calibration, higher power requirements, and more sophisticated processing. For example, as discussed by Preisendorfer (found in Mobley’s paper), the spectral properties are an aspect of light transport; such spectral data provides information to identify materials and determine the overall water quality. Multi- and hyperspectral techniques are capable of distinguishing materials and assessing water quality but leave open questions of what spectral resolution should be sufficient for achieving the desired level of performance in turbid-water imaging; for some applications, the usage of hyperspectral cameras may be limited to short stand-off distances, and for others additional calibration of the light source or image post-processing to correct for systematic errors may be required.

Looking at the studies, two main directions stand out for improving performance. One is laser-based imaging. The other is AI-based processing. But they work in different stages of the system. Laser-based systems, like laser-line scan and range-gated imaging, try to fix the problem at the source [85,86,87,88,89,90,94,95,96,97,98]. They control how light is used. By shaping or timing the illumination, they reduce backscatter and improve visibility. This works well in moderately murky water. It is useful for tasks like inspection, seabed mapping, and structural checks, where the setup can stay stable.

The key point is that these systems improve the measurement itself, not just the image afterward. That is why they can increase contrast and extend the usable range. But there is a cost. The hardware is more complex. Timing and alignment need to be precise. Power use is higher. And the systems can be sensitive to movement. Because of that, they are harder to use on small or fully autonomous platforms. In contrast, AI-based underwater imaging approaches [26,65,134,135,136,137,138,139,140,141,142,143,144,156,157,158,159,160,161,162,165] focus on computational recovery of contrast, color fidelity, structural detail, and semantic content from conventionally acquired images. Enhancement networks, depth estimation models, and semantic detection frameworks enable improved interpretability and autonomous decision-making in applications such as ecological monitoring, navigation, inspection, and object recognition. Still, the studies show a clear limit with AI. Its performance depends a lot on the input quality. If the image is poor, AI can only do so much. Changes between different water conditions also cause problems. And limited training data makes it harder to generalize.

So, AI cannot fully fix bad data. If the image is weak from the start, the results will also be limited. Because of this, newer work is moving in a different direction. Instead of relying only on AI, systems combine better acquisition with smarter processing. Some use physics-based models. Others combine laser-based imaging with AI methods. The idea is simple. Do not treat hardware and software separately. The best results come when both are designed together. Importantly, recent works that integrate physics-informed models or combine laser-based acquisition with AI-driven restoration suggest that the most robust performance is achieved when physical measurement design and computational intelligence are co-optimized rather than treated independently. Collectively, these findings indicate that performance improvement in underwater optical imaging follows two complementary but unequal strategies: acquisition-driven improvement through controlled illumination and measurement geometry, and computation-driven improvement through data-driven inference. The relative dominance of each strategy is strongly application-dependent, with industrial and metric tasks favoring acquisition optimization, while ecological monitoring and autonomous perception increasingly leverage AI-assisted interpretation within hardware-constrained platforms.

When the laser-based and AI-driven works discussed above are examined collectively through the structured comparisons presented in Table 2 and Table 3a, several system-level patterns become evident. First, systems that prioritize physical conditioning of the optical signal—such as those based on laser-line scanning and structured illumination [85,88,90]—consistently occupy the high-complexity, high-robustness region of Table 3a. These platforms demonstrate improved backscatter tolerance and geometric precision but require tighter calibration control, higher power budgets, and more rigid mechanical integration. In contrast, AI-centric systems summarized in works such as [1,11,83] predominantly improve perceptual or semantic performance while remaining dependent on conventional acquisition hardware. Table 2 and Table 3a reveal that these approaches tend to reduce hardware complexity but increase computational demand and training dependency, particularly when deployed on autonomous platforms with constrained onboard resources.

Table 2 also points out that the workload for calibration and the complexity of illumination are not treated the same in each sensing approach. Although lasers are used in both geometry and timing synchronization with high accuracy, AI-based RGB sensors still lack a complete set of the impacts of turbidity, incident angle, and attenuation. This is partly why it is so challenging to compare the results of different approaches and find the correlation between the increase in distance and the improvement rates in enhancement-based studies. Moreover, Table 3a shows that distance is more correlated with the acquisition method than post-processing. That is, those systems with an improvement in image acquisition are more likely to increase the working range, whereas those with image post-processing improve performance at a closer range only. Therefore, Table 1 and Table 2 provide the major observation of the surveyed literature that the best system performance is achieved with a system integration of image acquisition, sensor calibration, and image processing in a holistic framework rather than by viewing all sensing approaches in this paper as competing technologies. In fact, each sensor can be designed as a specific system that targets some scenarios, considering the varying working distance, clarity, power consumption, computation power, and data retention time. The proposed systems overview is the foundation of the in-depth system analysis at the system level presented in Section 3. Our system-level analysis investigates the modality-dependent trade-offs for complete pipelines for all types of sensors, going beyond the modality comparison in Section 2 by considering the underwater imaging systems rather than only their sensing components. As depicted in Figure 1, a typical underwater imaging system has five blocks, which include underwater image acquisition, pre-processing, data compression, data transmission, and surface data analysis. This illustrates that an overall system cannot be better than the worst component in it. According to Figure 1, the bottleneck of an imaging system does not have to exist in the sensing module, it could exist in every link of the system, since a failure at any stage of processing could lead to the failure of the whole image system pipeline. For example, image acquisition decisions, such as setting illumination, selecting the sensor or camera, or setting sensor configurations, can be major factors that affect the entire pipeline. The hardware chosen for acquiring the images may increase the complexity of the pre-processing of the images, the efficiency of the compression of the data, and the possibility of real-time transmission of data, which may be constrained by the data transmission bandwidth available to the network. The architecture of each system, in particular the processing location (i.e., onboard vs. surface or combined), sets the limits on each system. Systems that do some onboard pre-processing are built around bandwidth constraints in the downlink and in downstream surface processing; systems that do not onboard computation are designed around sensor-surface connectivity, often tolerating higher latency and/or bandwidth. Figure 1 shows a high-level architecture for these systems, which we use to frame the physical, computational, and communication trade-offs among existing systems in Section 3.1. Table 3a shows that the practical performance outcomes for underwater optics described in Table 3a depends (or does not depend) on how (if at all) each system performs data acquisition, calibration, processing, and transmission.

Table 5 shows that practical performance outcomes in the implementation of underwater imaging systems tend to depend more on system design, not just sensing modality. The shallow-water stereo systems in [263,264,267] can produce dense 3D reconstructions of the seafloor (with a context of stand-off distances, controlled imaging overlap, simultaneous capture, and precise calibration); however, this dense matching (and geometric correction) requires heavy post-processing and a small optical work space. Survey and BRUVS measurement systems [186] operating at close distances and small angles to ensure measurement accuracy show that even wide-field-of-view sensors in the water column are subject to optical distortion due to refraction through the water column. DSLR-based systems [184] provide greater optical and radiometric flexibility through the use of dome-port enclosures and lens calibration but also incur (mechanical and calibration) overhead in their implementation. In all the examples using human-operated systems, good geometric reconstruction, radiometric calibration, and signal-to-noise ratios result from post-deployment (and pre-deployment) procedures and are not a property of any single modality. The systems in Table 5 illustrate this: a rigidly mounted sensor in a repeatable configuration that is well calibrated is as good as a “good” imaging sensor in these contexts.

Literature reviews describe imaging systems capable of functioning in multiple acquisition modes (e.g., a video mode and a strobed/structured-light mode of the same sensor) [248]. In these instances, the modes are not presented separately; they are combined into a system-level category in either Table 5 or Table 6. This is because the review focuses on hardware systems rather than the configurations of modes (an intentional design decision, as this design-centric review compares system architectures that include, but are not limited to, sensors, lighting, calibration, and onboard computer design, instead of configurations more easily modifiable in different mission field settings). Of note are the inclusion and exclusion criteria that govern system-level synthesis. For example, in [256] no system or camera is proposed; rather, VIAME, a software analytics tool suite, is described, intended to be executed upon images acquired via existing systems. Consequently, the inclusion of [256] falls within the domain of the computational/analytics level of the processing pipeline, not as a system-level example of hardware, as this s not the work’s primary objective. In other words, ref. [256] serves as a good reference for how the machine learning frameworks, at the post-acquisition, analytics stage, can be paired to multiple sensor systems, once more drawing attention to the separation between imaging systems and imaging analysis.

Furthermore, ref. [181] does not describe a deployed system or camera; rather, it outlines a survey/comparative study of underwater imaging modalities. This is accomplished by classifying imaging systems into two basic imaging modes: passive systems, which employ only natural light (e.g., color, RGB, stereo and SfM), and active systems, which utilize active illumination (e.g., range-gated, laser scanning, structured-light, time-of-flight and polarimetric systems), highlighting the trade-off impact the choice of lighting source has not only upon the depth at which imaging systems can be deployed but upon performance and system complexity in turbid water. From a design perspective, ref. [181] does not describe a system-level demonstration and validation of a design, but rather provides an excellent reference for the advantages and disadvantages of each imaging mode as they apply to light-source use, size/weight and power requirements, as well as the intended use case for the system. The contribution of [181] is its system design-space mapping; it is not system-testing or validation work, thus serving as an excellent reference for an architectural discussion rather than a performance/system testing demonstration. Thus, ref. [181] is included as a reference for system design and hence does not appear in the system-level examples displayed in Table 5 and Table 6.

From the cross-tabulated summary in Table 6, we can see that the environmental conditions are a consistently predictable bottleneck that hinders performance of imaging in the underwater realm across the variety of sensing modalities. Specifically, we note that systems that utilize shallow water, near-field, or moderately clear water sensing conditions, like the stereo action-camera system [186] and DSLR-based photogrammetric platforms [184] in Table 6, generally deliver accurate geometric reconstructions, but only within very specific ranges and fields of view. As we begin to operate systems outside of these constraints, we frequently observe contrast-matching failure due to reduced SNR and degraded accuracy for geometric estimation owing to higher scattering and attenuation rates.

As systems are scaled to wider fields-of-view, these problems are more difficult to compensate for. Similarly, in more “natural” environments where ambient light cannot be managed or controlled, the above contrast reduction and reconstruction quality issues are further exacerbated. We can see from Table 6 that in deep-water, high-turbidity and/or long-range sensing environments passive systems are also bottlenecked by a signal loss that further reduces their image reconstruction performance due to a lack of contrast and high scattering. Active systems discussed in the literature that incorporate active illumination [89,90,91] have generally better performance and are more adept at operating in turbid conditions. In these high-turbidity sensing environments, high-quality reconstructions are achievable, but at the cost of additional bottlenecks, including power requirements, synchronization requirements and mechanical stability requirements.

We similarly observe that the gated imaging and line-scan laser systems, with comparable performance results to the other approaches mentioned here, are also particularly vulnerable to misalignment and timing jitter errors. In essence, environmental robustness must generally be exchanged for a more complex and less agile sensing system. For deployments that are expected to run for continuous operation or over extended periods of time, as in [181,186] in Table 6, there is a more complex set of constraints to satisfy that arises from varying environmental conditions as well as the mechanical instability of the sensing platform being deployed on. In these situations, the system requirements must be carefully balanced between power, calibration stability and data processing demands. Finally, other approaches discussed in this paper [65,134,135,136,137,138,139] utilize AI-based techniques to improve system performance on specific image reconstruction tasks such as depth estimation or image classification; however, they are fundamentally limited by the quality of the captured data. Should the imaging sensor fail to deliver sufficient data quality in the given environmental condition, then the resulting post-processing steps alone should not be expected to deliver a physically consistent reconstruction. Ultimately, such methods are no better than the quality of the data that are recorded and can only be effective as long as operating conditions are sufficiently stable and consistent with the training conditions.

Ultimately, we can see from our analysis of Section 2.1 and Section 2.2 that while imaging modality does play a role in overall system performance, it does not entirely determine it (Table 2 and Table 3a). Instead, the performance of a given modality is primarily contingent on whether its strengths address the environmental condition the underwater optical system will operate in (Table 2 and Table 3a). That is, the scattering and attenuation characteristics in a given body of water will determine which modalities will be effective (Table 2 and Table 3a). From Table 5 and Table 6, we can also see that, although the correct modality can be selected to operate in a given environmental condition, system performance only fully realizes the advantages of that choice when system design is also optimized to overcome environmental constraint limitations (Table 5 and Table 6).

Table 5 and Table 6 also reveal the impact individual design component selections can have on final system performance. We might even see a passive system with constraints work in a specific application [184,186]; however, this is highly dependent upon the environmental condition of the application at hand (Table 6). Active systems [89,90,91] can be more broadly applied and will perform at least as well if not better when operated under environmental conditions outside the aforementioned specific conditions (Table 5 and Table 6). This statement also applies to AI-based processing as compared to traditional image processing. We can see from Table 2 and Table 5 that AI provides a much better analysis of the quality of the environment [65,134,135,136,137,138,139]. This may suggest that while AI techniques will be most beneficial in assessing image quality, the effectiveness of using such AI techniques is still limited by the quality of the data itself, and it may be better to condition the data itself to produce higher image quality [134,135] in this manner. This is supported by [134,135], which show how AI can be utilized in an underwater environment to improve image interpretability. From Table 5, it can be seen that system calibration and sensor position also affect system performance.

In the case where a passive imaging system is not calibrated correctly, image interpretation will be different as the system is moved in relation to the target. These are similar conclusions to those drawn in Section 3, indicating that only by optimizing a system as a whole does the system achieve its maximum potential. We saw in that same section that optimizing each component of the system, that is, the illuminators, optics and algorithms, independently did not produce a system of a higher quality than if all components were optimized as a single system. Further, a co-designing approach to a system’s design will lead to a system that is more robust to changing operating conditions [90,91,186], rather than an individual-components approach, which can lead to complete system failure should one component operate in isolation (Table 6). Those failures include unaccounted-for imaging environments, system calibration drift over time, and vehicle capabilities that are not suitable for the application, e.g., use of a small AUV to perform a highly computational problem. A promising approach for improving underwater optical imaging is to focus more on the overall system integration rather than improving components independently. The bottom line is that the performance of a system depends on both the sensors used and how the components are integrated to perform a specific function.

## 6. Conclusions and Future Research

This paper has provided an overview of underwater imaging, emphasizing how systems-level engineering shapes environmental, mission, and design challenges. We have demonstrated that the system performance is a function of not only the capabilities of individual subsystems (e.g., sensors, algorithms, and optics), but also of the coupled system dynamics, ranging from the data acquisition method, calibration, and illumination to computational post-processing. Our analysis shows that, in many cases, optimizing for the system level may provide a more robust and effective engineering trade-off than optimizing only at the component level. System designs relying on passive RGB-compatible sensors are inherently limited by the ocean’s spectral optical transmission window. By contrast, complexity increases with the system-design complexity of active methods such as laser illumination or range gating, which require more careful engineering to obtain the SNR necessary for the desired image quality.

Computational post-processing techniques can enhance interpretability and downstream analysis, though these methods are constrained by the SNR of the data and the change in the optical signature of the target as a function of depth. In many cases, computational post-processing techniques are most advantageous in conjunction with data acquisition methods that can reduce the ill-conditioning of the inverse image formation problem and thereby reduce the need for ill-posed solutions. We have presented a mission-centric analysis to provide a summary of the state of the art in underwater imaging and provide a guide to the trade-offs in design choices to allow for optimal selection of end-to-end imaging systems for specific applications. It is our hope that this detailed systems analysis will guide engineering decisions in developing imaging systems with optimal configurations for particular applications. In the previous section, we discussed the recommended configurations for an underwater imaging system depending on the specific requirements of the mission and the capabilities of the existing sensor or optical component library. While our methodology provides a tool to help justify the choice of a particular imaging system for a mission or under a set of environmental conditions, we also call for greater rigor in the reporting of results, including the specification of the environmental parameters, the optical component selections, and the system design.

Future underwater optical imaging systems should increasingly adopt adaptive and integrated design strategies. Adaptive acquisition can dynamically modify illumination, exposure, wavelength, frame rate, or sensing modality according to changing water conditions and mission requirements. Optical–computational co-design offers further potential by jointly optimizing illumination, sensing hardware, and reconstruction algorithms rather than treating them as independent components. Multimodal systems combining RGB, polarization, laser-based, or spectral sensing may improve robustness across varying environments, while physics-informed AI can incorporate underwater image-formation constraints to improve generalization beyond specific training datasets. Future evaluation should also become more task-driven, assessing imaging systems not only through image quality metrics but through their ability to support downstream tasks such as detection, navigation, inspection, measurement, and ecological monitoring.

These parameters are essential to allow for a meaningful comparison and assessment of the performance and robustness of imaging systems in a given application. Going forward, we must continue to develop system designs that exploit recent advances in physics-based underwater image formation models, active illumination methods, machine learning approaches for both image restoration and target recognition, and system designs including system-level calibration for long-term stability under system perturbations due to motion, temperature and pressure, and bio-fouling. These system-level designs will also need to consider both calibration stability and power/resource considerations in practical applications. Finally, we call for greater inclusion of the deployment data for fielded applications and their robust, autonomous deployment capabilities, where appropriate, in the imaging system research literature. In summary, our analysis provides a systems-level framework for the design of underwater optical imaging systems which will allow these systems to meet the mission and environmental requirements of the imaging task at hand.

## Figures and Tables

**Figure 1:**
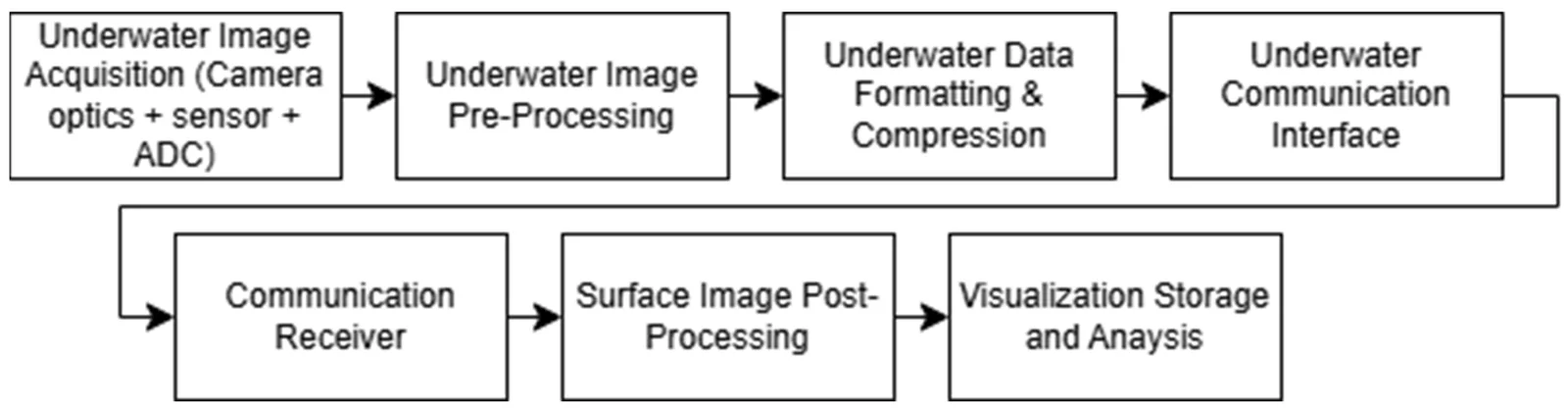
A generic underwater image pre-processing and post-processing pipeline.

**Figure 2:**
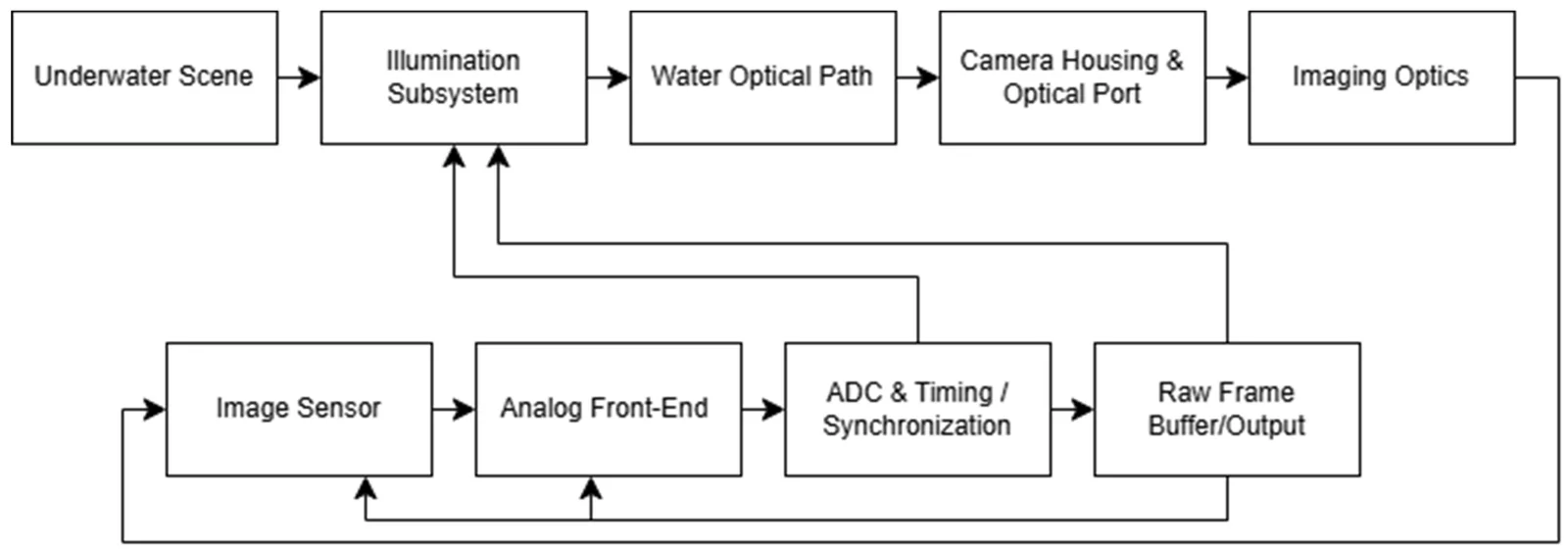
Underwater image acquisition function block.

**Figure 3:**
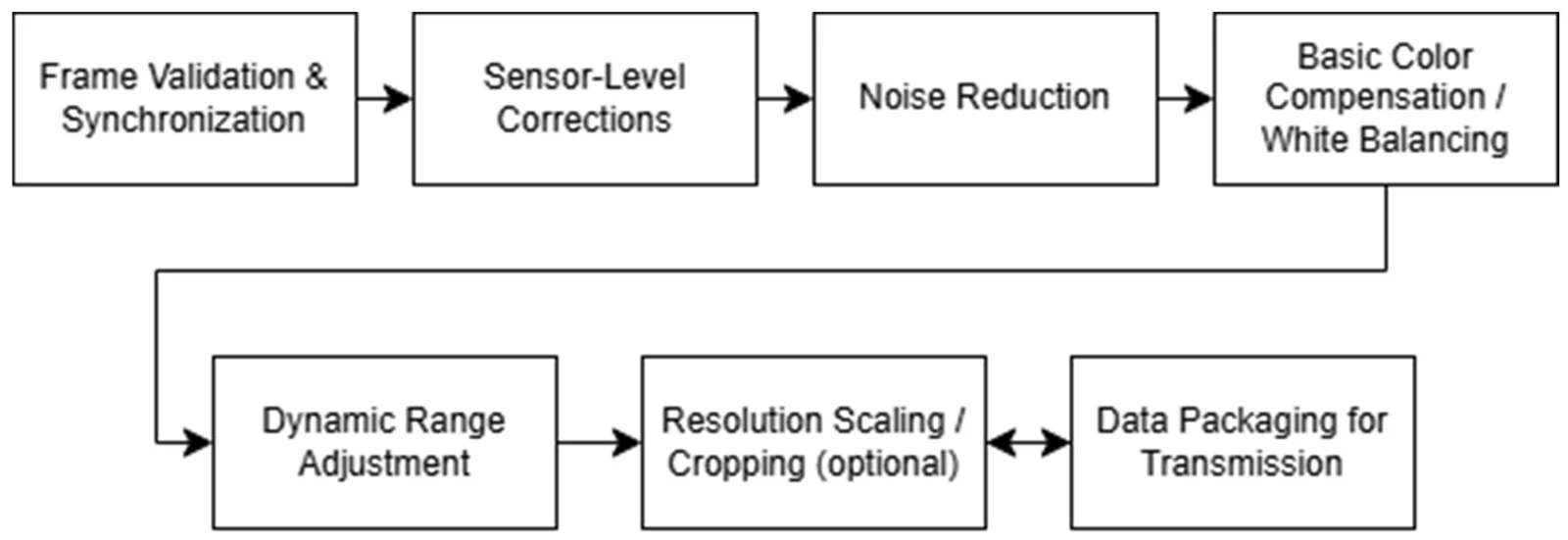
Underwater image pre-processing function block.

**Figure 4:**
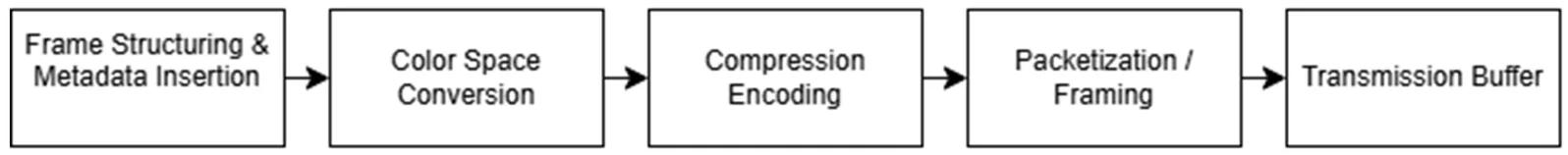
Underwater data formatting and compression function block.

**Figure 5:**
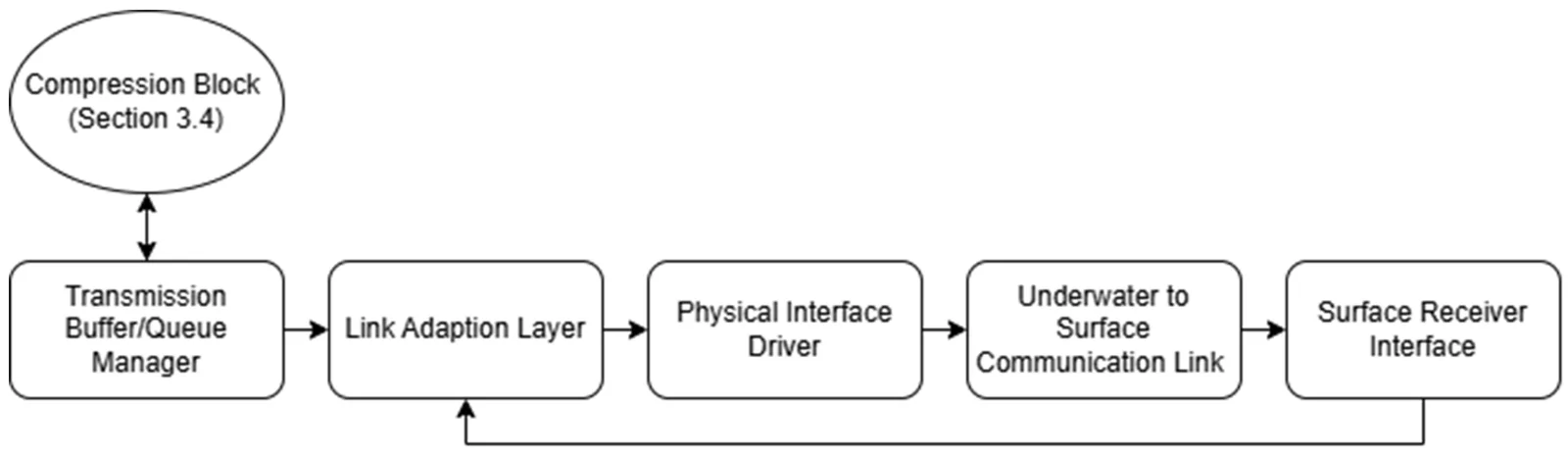
Underwater communication interface function block.

**Figure 6:**
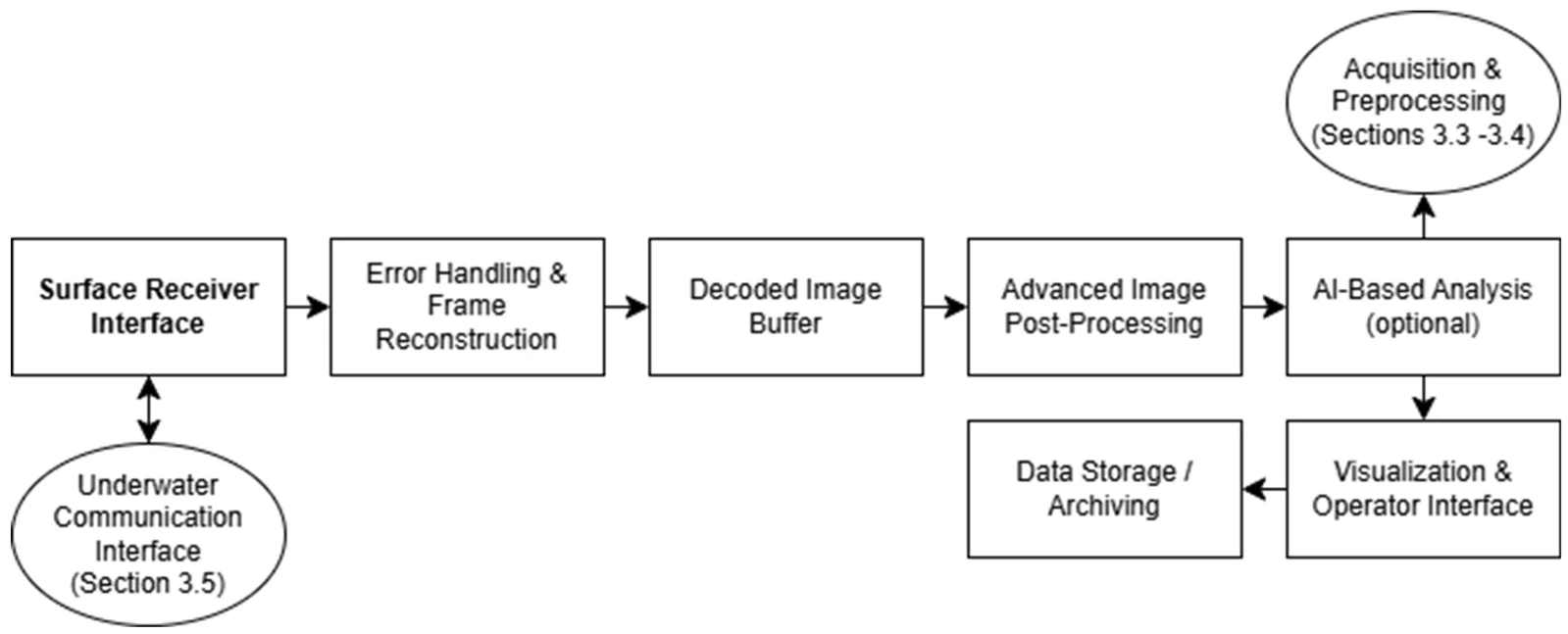
Surface image post-processing function block.

**Figure 7:**
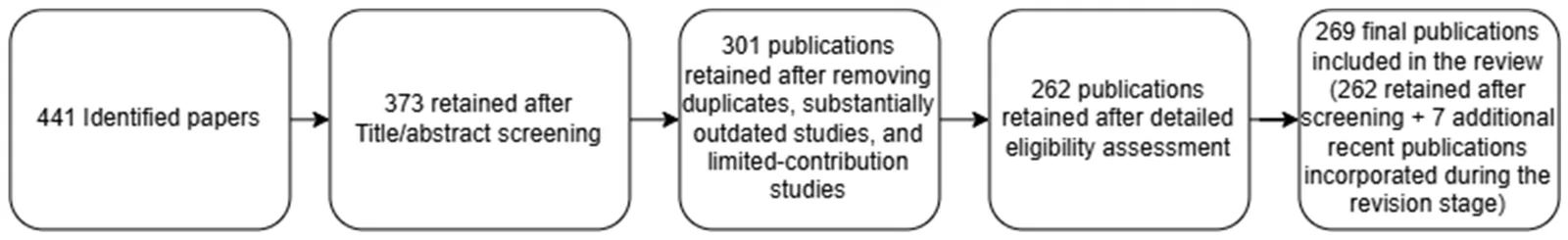
Literature identification, screening process and additional studies.

**Table 1:** Representative AI approaches for underwater optical imaging and their reported evaluation characteristics.

AI Approach	Representative Reference	Application	Metrics Explicitly Reported	Computational/Deployment Information	Main Limitation/Design Consideration
CNN/GAN-based	[140]	Underwater image enhancement	PSNR, SSIM, UIQM	17 MB memory; 25.4 FPS on NVIDIA Jetson TX2; designed for real-time robotic pre-processing	Performance degrades for severely degraded or texture-poor images; perceptual enhancement does not guarantee recovery of true pixel intensities
Transformer-based	[178]	Underwater image enhancement	PSNR, SSIM, UIQM, UCIQE, NIQE	15.436 M parameters; 0.031 s inference time in the authors’ comparative experiment; onboard deployment not demonstrated	Higher computational complexity than lightweight enhancement approaches
Hybrid/physics-informed	[150]	Underwater image enhancement	Evaluated on UIEB, UCCS and SQUID with quantitative image quality and complexity analyses	Combines physics-based priors, deep unfolding and learned regularization; onboard deployment not reported	Performance remains dependent on the suitability of the physical priors and learned regularizers
Self-supervised	[179]	Joint underwater depth estimation and image restoration	UIQM, UCIQE, PSNR, SSIM; depth evaluated using correlation and scale-invariant MSE	55 FPS reported; simultaneous depth and restored-image output; actual AUV/ROV onboard deployment not demonstrated	Requires monocular video during self-supervised training and relies on image-formation/view-synthesis constraints
Multimodal	[180]	RGB–sonar underwater object detection	mAP@0.5, mAP@0.5:0.95	53.6 FPS reported; Transformer-based RGB–sonar fusion; actual onboard robotic deployment not demonstrated	Cross-modal alignment, heterogeneous sensor characteristics, and relatively high parameter count

**Table 2:** Comparison of underwater optical imaging technologies and processing architectures.

Technology	Typical Processing Location	Real-Time Feasibility	Computational Demand	Strengths	Limitations
Conventional RGB Cameras	Onboard pre-processing & surface post-processing	Moderate	Low to moderate	Simplicity, low cost, widespread deployment	Strong sensitivity to turbidity and lighting
Laser-Based Imaging	Surface-based or distributed	Limited	High	Improved contrast and range performance	High power consumption and system complexity
Polarization Imaging	Surface-based processing	Limited	Moderate to high	Backscatter reduction and contrast enhancement	Experimental maturity and calibration sensitivity
Multispectral/Hyperspectral Imaging	Surface-based/offline	Rare	Very high	Material and habitat discrimination	Large data volume and heavy processing requirements
AI-Enhanced Optical Imaging	Onboard + distributed hierarchical systems	Increasing	Moderate to very high	Adaptive enhancement and semantic interpretation	Generalization, energy cost, hardware constraints

**Table 3:** (**a**) Comparative performance characteristics of major underwater optical imaging modalities. (**b**) Additional system-design characteristics of underwater optical imaging modalities.

(**a**)					
**Modality**	**Reference**	**Effective Range**	**Backscatter Robustness**	**Spatial** **Resolution**	**Hardware** **Complexity**
RGB	[26,61,72,195]	Short (0.5–5 m clear; <2 m turbid)	Low	High	Low
LLS	[85,86,87,88,89]	1–10 m (moderate turbidity)	High	Very high	Moderate
LRGI	[92,95,96,97,98,99]	2–15 m (moderate turbidity)	Very high	High	High
Polarization	[19,92,104,112,113,114,196,197]	0.5–3 m	Moderate	High	Moderate
Multispectral	[121,124,125]	<3 m	Low-moderate	Moderate	High
Hyperspectral	[126,127,129,130]	<2 m	Low	Moderate	Very high
AI Enhancement	[134,157,158,160,161,162,165,198]	-	Depends on input	Preserves structure	Low hardware
(**b**)					
**Modality**	**Typical Spectral Band**	**Turbidity Suitability**	**Power Demand**	**Calibration Burden**	**Platform Suitability**
RGB	Visible, mainly blue–green underwater	Low–moderate	Low–moderate	Low–moderate	AUV, ROV, fixed, diver
LLS	Typically visible laser, often blue–green	Moderate–high	High	High	ROV, larger AUV, fixed
LRGI	Typically blue–green pulsed laser	Moderate–high	High	High	ROV, larger AUV, fixed
Polarization	Visible	Moderate	Moderate	High	ROV, fixed, research platforms
Multispectral	Multiple selected visible/NIR bands	Low–moderate	Moderate–high	Moderate-high	ROV, fixed
Hyperspectral	Dense spectral bands	Low–moderate	High	High	ROV, fixed, research platforms
AI Enhancement	Depends on acquisition	Depends on input data	Moderate–high computational power	Depends on acquisition system	AUV, ROV, distributed systems

**Table 4:** Mission- and environment-driven selection matrix for underwater optical imaging modalities.

References	Depth/Ambient Light	Turbidity Suitability	Imaging Distance/Platform Speed	Suitable Modality	Design Rationale and Trade-Off
[26,61,72,187,195]	Shallow–moderate depth; useful ambient light or simple artificial illumination available	Low turbidity; relatively low attenuation and scattering	Short range; moderate–high platform speed	RGB/stereo imaging	Low hardware and processing complexity makes it suitable for mobile and real-time platforms; performance decreases rapidly as attenuation and backscatter increase
[104,110,111,112,113,114,115,116,196,197]	Shallow–moderate depth; natural or controlled illumination	Moderate turbidity; scattering is the dominant source of contrast loss	Short range; low–moderate speed; spatial polarimetry preferred for moving targets	Polarization imaging	Polarization cues can suppress backscatter and improve contrast, but require additional optical components and calibration; temporal polarization acquisition is more sensitive to scene/platform motion
[85,86,87,88,89,90]	Operational depth determined mainly by platform/housing; active illumination removes dependence on ambient light	Moderate–high turbidity; strong scattering/backscatter	Short–medium range; slow or well-controlled platform motion	Laser-line scanning (LLS)	Restricted illumination volume reduces backscatter and provides high spatial detail, but scanning geometry, alignment and platform stability are critical
[92,93,94,95,96,97,98,99]	Operational depth determined mainly by platform/housing; ambient illumination not required	Moderate turbidity; substantial backscatter and attenuation	Medium range; moving operation possible when precise laser–camera synchronization is maintained	Laser range-gated imaging (LRGI)	Temporal gating suppresses out-of-range backscatter and permits full-frame imaging, but requires pulsed illumination, precise timing and comparatively high system complexity/power
[100,101,102]	Shallow–deep deployment depending on platform; active illumination used	Clear–moderately clear water; performance increasingly limited by attenuation and scattering	Short–medium mapping/ranging distances; low–moderate platform speed	Underwater LiDAR	Provides direct range and 3D geometric information, but increasing turbidity and scattering strongly reduce useful range and measurement accuracy
[121,122,123,124,125,126,127,128,129,130]	Usually short-range operation; controlled illumination advantageous	Low–moderate turbidity; strong wavelength-dependent attenuation must be considered	Short range; stationary or relatively slow platform preferred	Multispectral/hyperspectral imaging	Provides spectral information for material, habitat and biological discrimination, but requires greater calibration, illumination control, storage and processing resources
[181]	Variable depth and ambient illumination; changing operating conditions	Variable turbidity, attenuation and scattering where one sensing channel may become unreliable	Variable range and platform motion depending on mission	Multimodal fusion (e.g., RGB–sonar or combined optical modalities)	Complementary sensing can improve robustness when a single modality becomes unreliable, but adds synchronization, calibration, sensor-integration and computational requirements

**Table 5:** System-level design attributes of representative underwater optical imaging systems.

Reference	Platform	Imaging Modality	Applications	Lighting	Image Acquisition
[263]	Fixed GoPro Hero 3 Black stereo camera kit deployed near seafloor	Stereo RGB still imagery for dense 3D reconstruction	Micro-bathymetry and seafloor roughness measurement	-	Synchronized stereo image pairs; wide-FOV lenses cropped and rectified; underwater calibration; dense stereo matching to generate high-resolution point clouds
[186]	Stereo-baited remote underwater video systems (BRUVSs) and pool-based rigs using paired GoPro GoPro Hero 3 Black action cameras	Stereo RGB videography	Fish length and body-size estimation in situ	-	Synchronized stereo video (1080p, 30 fps); fixed camera separation and convergence angle; measurements derived via stereo-photogrammetric software
[264]	Snorkeler-operated GoPro Hero 3 Black on coral reef flats (1–2 m depth)	Monocular RGB imagery used for structure-from-motion photogrammetry	Benthic habitat mapping, coral cover, coral health, holothurian counts (citizen science surveys)	Ambient natural light (shallow reefs, daylight surveys)	Continuous image capture at 2 Hz; ~60% image overlap; wide-angle lens; images processed offline using SfM to generate orthorectified 3D models
[265]	Tank-based experimental setup using GoPro Hero 3 Black	Monocular RGB imaging for metric photogrammetry calibration	Accuracy and stability assessment for underwater photogrammetric measurements	-	Controlled image sequences of calibration frame in water tank; multiple resolutions (7 MP, 12 MP); calibration and error analysis using check points
[267]	Diver-operated GoPro Hero 9 and SJCAM SJ4000 with standard underwater housings	Monocular RGB photography and videography for photogrammetric 3D reconstruction	Accuracy comparison of 3D point clouds; underwater object documentation	Ambient daylight (shallow water, cloudy conditions)	Oblique circular image capture with ~80% forward and ~70% side overlap; still images and video frames processed into 3D point clouds
[184]	Diver-operated underwater camera systems (DSLR, mirrorless, stereo, multi-GoPro rigs)	Monocular, stereo, and multicamera RGB photogrammetry	High-accuracy coral reef monitoring and deformation analysis over time	-	Images acquired at fixed camera-to-object distances (2 m, 5 m); calibrated using underwater geodetic reference networks; SfM processing for dense point clouds
[248]	Towed underwater platform (CamTrawl system)	Stereo optical imaging (visible light cameras)	Fish stock assessment and species identification	Artificial illumination	Continuous and triggered stereo image acquisition with camera–strobe synchronization during towed transects
[249]	Handheld infrared thermography used in marine ecology contexts (primarily intertidal/surface-access scenarios; underwater attenuation limitation noted)	Thermal infrared imaging (thermograms; pseudo-color rendering described)	Ecological applications: counting/tracking some marine mammals; intertidal thermal ecology (organism/surface temperature variability)	Passive thermal emission (infrared radiation from objects)	Emphasizes calibration/ground-truthing needs; key parameters include emissivity and environmental effects; notes infrared is rapidly attenuated by seawater (limiting true underwater use)
[250]	Small autonomous vessel/ASV (R/V Philos system)	LWIR (8–15 μm) camera imagery compared against optical; semantic segmentation on LWIR	Maritime object detection/scene understanding under extreme lighting and weather; supports navigation/avoidance	Passive thermal (LWIR is independent of visible light; based on thermal emission)	FLIR LWIR cameras (reported 640 × 512, 75° FOV); dataset of LWIR images created; segmentation tested with WaSR and UNet
[251]	LoCO AUV (low-cost, open, vision-guided AUV; dual-camera system)	Dual-camera vision payload (optical cameras)	Vision-guided autonomy demonstrations, including gestural control and diver following (and motion-based communication)	-	Designed to run vision-based algorithms (often deep learning for perception); detailed capture settings are not specified in the shown sections
[185]	Custom-built compact underwater imaging system with new underwater housing (rated for shallow seawater field deployment; handheld or tethered operation)	Polarization imaging and multispectral imaging using a bio-inspired X3 vertically stacked sensor and a polarized monochrome sensor (Sony IMX250-MZR via FLIR Blackfly S)	Underwater geolocalization using celestial polarized light; scientific underwater data collection; field deployment demonstrated in Hawaii	-	USB3 image acquisition via FPGA-based camera system; raw frames digitized (14-bit), buffered, and streamed to PC; custom Python-based camera control and data collection software (MantisCam); HDF5 data storage
[252]	Monocular camera + single active light setup in an indoor tank (used for underwater relative localization experiments)	Monocular optical camera (FLIR camera in pressure housing); light appears as a bright image feature	Learning-based relative underwater pose/location estimation using a single light source	-	Images collected in a tank; light-feature image processing pipeline; networks (FNN/CNN) trained; exposure time used as an additional input feature for CNN (feature concatenation discussed)
[93]	UTOFIA underwater range-gated camera system deployed from poles, frames, ROVs, USVs, and benthic sledges; integrated camera + laser in pressure housing rated to 300 m	Range-gated optical imaging with time-of-flight (ToF) monochrome CMOS sensor, providing 2D intensity images and real-time 3D measurements	Aquaculture monitoring, seafloor mapping, marine litter identification, structure inspection, fish sizing, and tracking	Artificial lighting: actively Q-switched Nd:YVO_4_ pulsed laser at 532 nm synchronized with camera shutter; system designed to be largely independent of natural light	Synchronized nanosecond laser pulses and gated shutter; range scanned by shifting laser–camera timing; ToF sensor (up to 1280 × 1024) acquires multiple gated exposures per frame to construct depth slices and real-time 3D at ~10 Hz; data processed via onboard FPGA and GUI for visualization and storage
[255]	IPAX (In situ Plankton Assemblage eXplorer): autonomous, self-contained underwater imaging instrument in cylindrical housing rated to 100 m, built around Raspberry Pi Zero W and Sony IMX219 camera	Monocular RGB optical imaging using Raspberry Pi-compatible Sony IMX219 sensor with interchangeable M12 lens (6 mm F2.0 used)	In situ zooplankton observation, diversity assessment, phototactic behavior analysis, and enumeration of major taxonomic groups	Artificial lighting: onboard LED flash unit (10 CREE XPE2 LEDs); deployments used white LEDs (6500 K) and far-red LEDs (730 nm) to study phototaxis	Video recorded at 30 fps, 1640 × 1232 px, exposure ~500 μs, ISO 200; LED strobed at 50% duty cycle during acquisition; programmed to record 30-s videos every 10 min from 18:00 to 07:00 over multiple nights; data stored on onboard SD card
[253]	SeeStar modular subsea imaging system composed of camera, battery, and LED modules (each rated to 300 m), deployable autonomously or tethered on ROVs, AUVs, landers, piers, and moorings	RGB still and video imaging using GoPro HERO cameras (Hero 2 and Hero 3/3+) integrated in pressure housing	Mid- to long-term ecosystem monitoring, fish surveys, jellyfish observation, benthic ecology studies, and general biological time-series imaging	Artificial lighting: dedicated LED lighting module (5-LED or 9-LED configurations up to ~3675 lumens) synchronized with camera triggering	Custom controller triggers GoPro for stills or video; programmable interval sampling; images stored onboard camera; examples include video capture on landers (~100 m) and periodic still imaging every 20 min for months during Antarctic benthic deployments
[254]	PlasPi marine camera system: Raspberry Pi Zero W–based autonomous camera in plastic pressure housing rated to 150 m, deployable on tripods, landers, CTDs, or towed platforms	Monocular RGB still imaging using Raspberry Pi Camera Module V2 (3280 × 2464 pixels)	Seafloor time-series monitoring, coral reef observation, benthic community change detection, “housekeeping” monitoring of marine instruments (e.g., optodes)	Artificial lighting: onboard LED illumination (three LEDs), triggered with each image	Python-controlled interval photography (default 1 image per 60 s); LED flash fired for each frame; images timestamped and stored on onboard SD card; field deployments collected ~20,000 images from 10 cameras at ~100–150 m depth; capture intervals ranged from 5 s (towed) to 1–5 min (static time series)
[256]	VIAME (Video and Image Analytics for Marine Environments): open-source software platform processing imagery from AUVs, stationary camera arrays, and towed vehicles (external hardware not defined by authors)	Optical RGB image and video streams provided by upstream systems (sensor hardware not specified)	Automated detection, classification, tracking, and abundance estimation of marine organisms for fisheries assessment	(lighting not specified; dependent on upstream data sources)	Accepts image lists, video files, or streaming inputs; processes frames through configurable pipelines supporting stereo matching, object detection, classification, and spatiotemporal tracking
[187]	Deep-sea stereo camera system integrated on commercial Apache ROV, consisting of two digital cameras in pressure bottles daisy-chained to an onboard compute bottle (Intel NUC), validated by pressure-tank tests and field trials offshore Marseille	Stereo RGB optical imaging using two Point Grey Grasshopper2 cameras (Sony ICX285 CCD) with 4.8 mm lenses, providing dense depth via stereo triangulation	Navigation, mapping, object recognition, manipulation support, inspection, and intervention for intelligent ROV operations (DexROV project)	Artificial lighting (deep-sea illumination fully controlled by ROV lights; paper explicitly states deep-sea visibility relies on artificial light)	Hardware-synchronized stereo capture via FireWire (1394b); baseline 0.3 m; images rectified using pinax refraction model; onboard stereo processing on Intel NUC; depth from SAD and deep learning stereo; validated in tank (1500 m equivalent) and sea trials (30 m depth)
[181]	Not a hardware platform—review of multiple underwater optical imaging systems and methods	Reviews active and passive optical modalities, including range-gated imaging, laser scanning, structured light, polarimetric imaging, TOF, stereo vision, monocular vision, and structure from motion	Aquaculture monitoring, underwater ecosystem observation, seafloor mapping, underwater archaeology, infrastructure inspection, robot navigation, and biomass exploration	Covers both artificial illumination (laser, LED) and natural light, depending on the method; lighting is discussed comparatively, not fixed	No unified acquisition system; summarizes acquisition strategies across surveyed works (e.g., synchronized laser–camera gating, stereo capture, monocular imaging, multiview SfM, SPAD-based TOF)
[257]	OpenROV 2.8–based assistive robotic system tethered to surface PC, integrating camera, LED lights, scaling laser, BeagleBone Black, Arduino, and MATLAB GUI for real-time control and processing	Monocular RGB video imaging using onboard USB webcam (max 720p at 30 fps) streamed to surface computer	Real-time detection and localization of invasive red lionfish to assist divers in targeted removal operations	Artificial lighting: onboard LED light board (4 LEDs, ~200 lm total); experiments conducted under ROV-controlled illumination	Live video streamed through 90 m tether; camera frames received every ~0.8 s; maximum 30 fps at HD 720p; frames processed in real time on surface PC via CNN; joystick-controlled navigation with synchronized lighting and camera tilt; field trials conducted at artificial reefs in the Gulf of Mexico

**Table 6:** Cross-study synthesis of environmental conditions, fundamental limitations, and system-design bottlenecks.

Reference	Environment	Fundamental Limitation	Design Bottleneck	Real-Time Processing	Offline Processing
[263]	Shallow seafloor imaging in clear water at short stand-off distances (≈0.2–1 m); controlled lighting conditions required	Light scattering in the water column degrades image sharpness and reduces reliable pixel matching, especially over homogeneous or low-texture surfaces	Very short operational range and fixed short stereo baseline (3.5 cm) constrain achievable depth resolution and limit applicability to close-range, high-texture scenes	-	✓
[186]	Shallow reef environments using stereo-BRUVS, with measurements restricted to targets within 5–7 m and limited angles to the optical axis	Optical distortion and accuracy degradation increase with distance and angle due to water-column effects and wide-angle (fish-eye) lenses	Strict constraints on camera–target distance (<5 m) and viewing angle (<25°) are required to maintain acceptable measurement accuracy, reducing effective sampling volume	-	✓
[264]	Shallow coral reef flat (1–2 m depth) surveyed by snorkel in clear tropical reef conditions	Limited image resolution and capture geometry leads to reduced detection of fine features and lower species diversity estimates compared with in situ visual surveys	Reliance on high image overlap and substantial post-processing for dense 3D reconstructions limits effective depth range (>5 m) and increases offline workload	-	✓
[265]	Controlled shallow underwater environment (water tank, 1–2 m camera-to-target distance) with clear water and artificial illumination	Refraction at the air–housing–water interfaces and sensitivity of calibration parameters to camera–housing alignment introduce systematic geometric errors	Requirement for repeated in situ calibration using a 3D calibration frame and careful camera mounting limits rapid deployment and operational flexibility	-	✓
[267]	Very shallow, clear-water underwater environment (≈1 m depth) under calm sea conditions; controlled platform to stabilize object and targets	Image quality and reconstruction accuracy are fundamentally constrained by water clarity, surface motion, and limited feature distinctiveness in smooth or homogeneous surfaces	High dependence on very high image overlap (≈80% forward, 70% side) and extensive post-processing (SfM, dense matching, manual filtering) increases computational load and limits scalability to larger or more dynamic environments	-	✓
[184]	Shallow coral reef environment (~12 m depth) surveyed by scientific divers over a small spatial extent (~5 × 5 m^2^) under clear-water conditions	Refraction at housing ports and residual systematic optical errors persist even after self-calibrating bundle adjustment, with errors increasing with camera–object distance	Achieving millimeter-level accuracy requires dense image networks, geodetic control points, careful camera–housing alignment, and extensive post-processing, limiting scalability and rapid deployment	-	✓
[248]	Arctic and sub-Arctic marine environments, including ice-covered seas (aerial surveys over sea ice) and pelagic midwater environments (trawl-based imaging)	Thermal contrast variability between targets and background (ice temperature, weather, animal size) and light attenuation underwater constrain reliable detection and classification	Dependence on stable environmental conditions, high power and payload capacity, and large data volumes requiring robust sensor integration and processing pipelines	✓	✓
[249]	Maritime surface environment under varying illumination, weather, and sea-state conditions	Thermal contrast between objects and background is scene- and weather-dependent, limiting detectability under low temperature differentials	Reliable detection requires sensor fusion (LWIR with optical/radar) and environment-specific tuning, increasing system complexity	✓	✓
[250]	Coastal and inland maritime environments with glare, fog, low-light, and nighttime conditions	LWIR imagery provides limited spatial detail and texture compared to visible imagery, constraining fine-grained classification	Lower image resolution and reduced semantic detail necessitate deep learning models and large annotated datasets	✓	✓
[251]	Pool, lake, and open-ocean deployments using the LoCO vision-guided AUV; platform rated to 100 m depth	Underwater vision performance depends on camera capability, while higher-performance cameras impose greater onboard computational load	Balancing camera performance, computational resources, power/endurance, cost, and modular sensor integration on a compact AUV	✓	-
[185]	Shallow seawater environments (field deployment in Hawaii) using tethered and handheld operation with artificial housing and dome port	Underwater polarization and multispectral measurements are fundamentally affected by light attenuation, scattering, and stress-induced birefringence in optical ports	Achieving reliable polarization/multispectral data requires bulky custom housings, careful optical alignment, thermal management, and high power/computation support, limiting portability and rapid deployment	✓	✓
[252]	Controlled indoor-tank environment using a monocular camera and a single active light for relative underwater localization	Optical detection range is affected by water clarity and ambient illumination; a single light provides limited direct geometric information	Reliable estimation depends on light-feature extraction and FNN/CNN generalization across position and camera-exposure variations; open-water performance was not demonstrated	-	✓
[93]	Coastal and open-ocean environments across variable turbidity and depth, using active laser illumination	Volume backscatter and light attenuation fundamentally limit optical range even with pulsed laser illumination	Increased system complexity, power demand, and synchronization requirements between laser and sensor constrain deployment scalability	✓	✓
[255]	Shallow coastal and freshwater environments for in situ zooplankton imaging at very short working distances	Limited depth of field and strong dependence on target proximity and illumination geometry	Small imaging volume and sensitivity to focus and alignment restrict applicability to localized, close-range observations	-	✓
[253]	Subsea deployments from shallow to mid-depth (≤300 m) using autonomous or tethered platforms	Reduced field of view and optical distortion caused by flat ports and wide-angle lenses underwater	Limited manual camera control and low-light performance constrain image quality and quantitative analysis	-	✓
[254]	Shallow-to-mid-depth seafloor environments (<150 m) for long-term time-series monitoring	Illumination fall-off and scattering restrict usable imaging range and spatial coverage	Low-cost hardware necessitates conservative illumination and exposure settings, limiting resolution and contrast	-	✓
[256]	Diverse marine environments using AUVs, towed systems, and stationary cameras	High variability in image quality and scene conditions limits generalization of automated analysis	Massive data volumes require extensive offline processing pipelines and curated training data	-	✓
[187]	Deep-sea environments with controlled artificial lighting and good inherent visibility	Stereo reconstruction accuracy fundamentally constrained by baseline length and quantization error	Large baselines and pressure-resistant housings increase system size, cost, and integration complexity	✓	✓
[181]	-	-	-	-	-
[257]	Coastal reef environments with low visibility and dynamic backgrounds	Optical clutter, variable lighting, and motion reduce robustness of visual object detection	Real-time deep learning inference requires high computational resources and stable illumination, limiting onboard autonomy	✓	✓
[231]	Shallow to mid-depth marine environments under varying turbidity and illumination	Backscatter and wavelength-dependent attenuation degrade image contrast and effective range	Performance gains rely on computationally intensive enhancement and learning-based methods, limiting real-time use	-	✓
[4]	Deep and shallow underwater archaeological sites surveyed using ROVs, AUVs, and towed platforms under low-visibility conditions	Rapid attenuation of visible light, low contrast, and strong backscatter fundamentally limit optical imaging range and the ability to capture large structures in a single frame	Achieving site-scale coverage requires extensive redundant imaging, careful lighting geometry, and computationally intensive post-processing (photomosaics and 3D reconstruction), limiting quantitative accuracy and operational efficiency	-	✓
[9]	Both **shallow water (natural light)** and **deep water (artificial light)** conditions for AUV-based optical target detection	Light scattering causes image blurring and color attenuation; traditional scattering-model restoration performs well only under natural light, not under artificial illumination	Achieving reliable restoration in both lighting regimes requires **parameter optimization** and additional computation to balance scattering models, limiting real-time performance in diverse environments	-	✓
[10]	Controlled turbid-water environment (large test tank) with artificial illumination and fixed camera–target geometry	Strong volume scattering and attenuation in turbid water fundamentally limit contrast under conventional floodlighting, especially when artificial illumination is used	Confocal imaging requires precise projector–camera calibration and long acquisition times due to sequential scanning, limiting real-time deployment and operational scalability	-	✓
[19]	Shallow marine environments (field experiments in the Red Sea at depths ~10–26 m) using natural illumination and diver-operated imaging	Visibility fundamentally limited by veiling light (path radiance) caused by scattering, which increases with range and dominates over blur and attenuation at moderate distances	Requires multiple polarization-filtered acquisitions, stable illumination between frames, and careful optical housing design (external polarizer, dome port) to avoid birefringence and polarization distortion	-	✓
[86]	Controlled clear-water tank environment with close range (≤~1 m) measurements for high-resolution laser scanning	Refraction at air–glass–water interfaces and attenuation plus backscatter limit laser stripe contrast and measurement accuracy as range increases	Achieving accurate 3D reconstruction demands precise refraction compensation, galvanometer calibration, and careful alignment of laser/camera subsystems, restricting use to short distances and offline workflows	-	✓

**Table 7:** Literature search strategy and retrieval results.

Source	Search Approach	Main Search Terms	Search Period	Publication Coverage
Scopus	Primary bibliographic search/structured search of indexed journal articles and conference publications	“Underwater optical imaging”, “underwater vision”, “optical system design”, “sensor configuration”, “attenuation”, “scattering”, “turbidity”, “laser imaging”, “polarization imaging”, “multispectral imaging”, “hyperspectral imaging”, “fluorescence imaging”, “marine ecology”, “seabed mapping”, “ROV imaging”, “AUV imaging”, “underwater navigation”	From 2 February 2026 for approximately two months	No strict lower publication-date limit; emphasis on recent literature with inclusion of seminal and foundational earlier studies
Google Scholar	Complementary search source/broader keyword-based scholarly search	Same thematic groups, using combinations of the search terms listed for Scopus	From 2 February 2026 for approximately two months	No strict lower publication-date limit; emphasis on recent literature with inclusion of seminal and foundational earlier studies

## Data Availability

No new data was created or analyzed in this study. Data sharing is not applicable to this article.

## Abbreviations

The following abbreviations are used in this manuscript:

ADC	Analog-to-Digital Converter
AFE	Analog Front End
AI	Artificial Intelligence
ASV	Autonomous Surface Vehicle
AUV	Autonomous Underwater Vehicle
CCD	Charge-Coupled Device
CMOS	Complementary Metal–Oxide–Semiconductor
CNN	Convolutional Neural Network
DSLR	Digital Single-Lens Reflex
FLIR	Forward-Looking Infrared (Commercial Camera Systems)
FOV	Field of View
FPGA	Field-Programmable Gate Array
GPU	Graphics Processing Unit
LED	Light-Emitting Diode
LiDAR	Light Detection and Ranging
LLS	Laser-Line Scan
LRGI	Laser Range-Gated Imaging
LWIR	Long-Wave Infrared
RGB	Red, Green, Blue
ROV	Remotely Operated Vehicle
SfM	Structure from Motion
SNR	Signal-to-Noise Ratio
ToF	Time of Flight
USV	Unmanned Surface Vehicle
UOI	Underwater Optical Imaging
VSF	Volume Scattering Function
